# Plasma membrane‐localized plant immune receptor targets H^+^‐ATPase for membrane depolarization to regulate cell death

**DOI:** 10.1111/nph.17789

**Published:** 2021-10-30

**Authors:** Hye‐Young Lee, Ye‐Eun Seo, Joo Hyun Lee, So Eui Lee, Soohyun Oh, Jihyun Kim, Seungmee Jung, Haeun Kim, Hyojeong Park, Sejun Kim, Hyunggon Mang, Doil Choi

**Affiliations:** ^1^ Plant Immunity Research Center Seoul National University 1 Gwanak‐ro, Gwanak‐gu Seoul 08826 Korea; ^2^ Department of Agriculture, Forestry and Bioresources Plant Genomics and Breeding Institute Seoul National University 1 Gwanak‐ro, Gwanak‐gu Seoul 08826 Korea

**Keywords:** *Capsicum annuum*, cell death, coiled‐coil domain, innate immunity, *Nicotiana benthamiana*, plant NLR, plasma membrane H^+^ ATPase

## Abstract

- The hypersensitive response (HR) is a robust immune response mediated by nucleotide‐binding, leucine‐rich repeat receptors (NLRs). However, the early molecular event that links activated NLRs to cell death is unclear.
- Here, we demonstrate that NLRs target plasma membrane H^+^‐ATPases (PMAs) that generate electrochemical potential, an essential component of living cells, across the plasma membrane. CC^A^309, an autoactive N‐terminal domain of a coiled‐coil NLR (CNL) in pepper, is associated with PMAs. Silencing or overexpression of PMAs reversibly affects cell death induced by CC^A^309 in *Nicotiana benthamiana*.
- CC^A^309‐induced extracellular alkalization causes plasma membrane depolarization, followed by cell death. Coimmunoprecipitation analyses suggest that CC^A^309 inhibits PMA activation by preoccupying the dephosphorylated penultimate threonine residue of PMA. Moreover, pharmacological experiments using fusicoccin, an irreversible PMA activator, showed that inhibition of PMAs contributes to CNL‐type (but not Toll interleukin‐1 receptor NLR‐type) resistance protein‐induced cell death.
- We suggest PMAs as primary targets of plasma membrane‐associated CNLs leading to HR‐associated cell death by disturbing the electrochemical gradient across the membrane. These results provide new insight into NLR‐mediated cell death in plants, as well as innate immunity in higher eukaryotes.

## Introduction

Plants rely on the innate immune system to defend against invading pathogens. One of the major immune responses is provoked by the perception of pathogenic effectors via intracellular immune receptors, designated as nucleotide‐binding and leucine‐rich repeat proteins (NLRs) (Maekawa *et al*., 2011). Activation of NLRs often accompanies rapid localized cell death, a phenomenon known as the hypersensitive response (HR). Plant NLRs are subdivided into two major classes depending on their N‐terminal domain: coiled‐coil NLRs (CNLs) and Toll interleukin‐1 receptor NLRs (TNLs) (Jones *et al*., 2016). Recent discoveries have shed light on the molecular events underlying NLR activation and NLR‐mediated cell death. Like bacterial and mammalian TIR domains, the plant TIR domain in TNL cleaves the oxidized form of NAD (NAD^+^), leading to cell death signal transduction (Horsefield *et al*., 2019; Wan *et al*., 2019). However, structural and biochemical characterization of HopZ‐activated resistance 1 (ZAR1) in CNL revealed that activated ZAR1 assembles a pentameric wheel‐like resistosome, and the N‐terminal α‐helix of the activated resistosome forms a funnel‐shaped structure, which is essential for ZAR1‐mediated cell death (Wang *et al*., 2019). Although a recent study reported that the ZAR1 resistosome acts as a calcium‐permeable cation channel, and the Glu11 carboxylate ring in the N‐terminal α‐helix is essential for this channel activity and cell death (Bi *et al*., 2021), the early molecular events that occur during the onset of cell death by activated NLRs remain elusive.

The NLR super gene family is present throughout plant species from mosses and liverworts to gymnosperms and angiosperms (Ortiz & Dodds, 2018). Genome‐wide comparative analysis has revealed that NLRs are divided into several groups according to their domain composition and sequence similarity. In Solanaceae, most of NLR super family members fall into 14 subgroups (Seo *et al*., 2016). Among them, a distinct subgroup of NLRs is classified as ANL (ancient and autonomous NLRs) which is well conserved in seed plants and triggers HR‐associated cell death via its own CC domains independently of known helper NLRs (Lee *et al*., 2021). ANL309 is a pepper (*Capsicum annuum*) NLR belonging to the ANL group and has potential to form multimeric NLR complexes (Supporting Information Fig. S1). Recently, we showed that CC^A^309 is a minimal segment of ANL309 (1–120 amino acids) which induces cell death similar to that induced by *R* proteins and is localized at the plasma membrane (PM) where many NLRs target after activation (Lee *et al*., 2021). Therefore, deciphering the mechanism underlying CC^A^309‐mediated cell death gives a clue to understand how activated NLR triggers HR cell death.

Many of the earliest cellular responses to pathogen infection are regulated by PM‐localized enzymes and ion channels (Boller & Felix, 2009). PM H^+^‐ATPases (PMAs), which pump out protons from the cytosol into the extracellular space, are a major player in creating a proton gradient and negative membrane potential. This electrochemical gradient is essential for transporting water, ions and nutrients across the PM. The activity of PMAs is turned on and off dynamically during plant immune responses, and multiple pathogens target PMAs during pathogenesis (Elmore & Coaker, 2011). The Avr5 effector of the fungal pathogen *Cladosporium fulvum* is recognized by the cognate *R* gene, *Cf5*, in tomato cell lines, resulting in activation of PMAs, followed by acidification of the extracellular matrix (Vera‐Estrella *et al*., 1994). In addition, apoplastic acidification by an activated PMAs is observed during interaction between barley Mla3 and AvrMla3 of powdery mildew fungus *Blumeria graminis* f. sp. *hordei* (*Bgh*) (Zhou *et al*., 2000). Intriguingly, apoplastic alkalization caused by the barley–*Bgh* interaction is mediated by different *R* genes (Elmore & Coaker, 2011). Although alkalization of the apoplast precedes cell death in many elicitor‐ or pathogen‐treated cells, it is not clear how the recognition event affects activity of PMAs and its effect on other cellular responses such as Ca^2+^ influx, the oxidative burst and cell death.

In this study, we identified *Nicotiana benthamiana* PMAs (NbPMAs), including NbPMA1, 3 and 4, as primary targets of PM‐associated CNLs to facilitate cell death. CNLs are associated with, and inhibit, the activity of PMAs, resulting in alkalization of the extracellular matrix, leading to membrane depolarization. We found that 14‐3‐3, an activator protein of PMAs, negatively affects the association between PMA and autoactive CC^A^309, and that the latter associates preferentially with inactive nonphosphorylated PMA rather than active phosphorylated PMA. Moreover, we observed that a fungal phytotoxin fusicoccin (FC), an activator of PMA, inhibits PM‐localized CC‐ or NLR‐meditated cell death as well as resistance. These results suggest that PMAs are key proteins that link the onset of cell death with other defense responses in PM‐associated CNLs.

## Materials and Methods

### Plant materials and growth conditions

*Nicotiana benthamiana* and *Arabidopsis thaliana* plants were grown in a walk‐in growth chamber at 25°C under a 16 h : 8 h (day : night) cycle. Four‐week‐old plants were used for *Agrobacterium*‐mediated transient expression. *rps2* (CS6196) mutants in the Col‐0 background were obtained from the Arabidopsis Biology Resource Center (https://abrc.osu.edu/).

### Transient gene expression in *N. benthamiana*

For *in planta* transient overexpression, cells of *Agrobacterium tumefaciens* strain GV3101 carrying the desired binary vector clones were grown overnight at 28°C in LB medium containing appropriate antibiotics. Cells were harvested by centrifugation at 1500 ***g*** for 10 min and resuspended in infiltration buffer (10 mM MES (2‐[*N*‐morpholino] ethanesulfonic acid), 10 mM MgCl_2_ and 150 μM acetosyringone, pH 5.6). Resuspended cells were incubated for 1–3 h at room temperature and pressure‐infiltrated into *N. benthamiana* leaves using a 1 ml needless syringe. To visualize cell death, leaves were photographed 3–5 d after infiltration using the Cy5 and Cy3 channels of the Azure 400 (Azure Biosystem, Dublin, CA, USA).

### Constructs

Clones for expression of epitope‐tagged proteins were generated using a ligation‐independent cloning method (Oh *et al*., 2010). The inserts encoding *NbPIP2;1* (Niben101Scf06928g00008.1), *Nb14‐3‐3* (Niben101Scf02367g04001.1), *NbPMA1* (Niben101Scf00593g01002.1), *NbPMA3* (Niben101Scf07395g00031.1) and *NbPMA4* (Niben101Scf03979g02010.1) were amplified from *N*. *benthamiana* cDNA and cloned into pCAMBIA2300‐LIC vectors harboring the cauliflower mosaic virus 35S promoter with an N‐terminal 6 × HA, C‐terminal eGFP or C‐terminal 3 × FLAG tag. For expression of fragments of NbPMA3, NbPMA3‐N (residues 1–64), NbPMA3‐M (residues 305–650) and NbPMA3‐C (residues 846–956) were cloned into the pCAMBIA2300‐C‐eGFP‐LIC vector (Zuo *et al*., 2000). For inducible expression, CC^A^309 (nucleotides 1–420) was cloned into the XVE‐DC‐6 × MYC vector by gateway cloning (Invitrogen). To generate single gene‐silenced plants, the gene‐specific fragment of *NbPMA3* (nucleotides 308–474) or 14‐3‐3 (nucleotides 54–338) was cloned into TRV2‐LIC vector. For cosilencing of *NbPMA*s (PMA1/2/3 or PMA1/2/3/4), the fragments of *NbPMA1* (nucleotides 4–204), *NbPMA2* (nucleotides 202–401), *NbPMA3* (nucleotides 402–601) or *NbPMA4* (nucleotides 599–798) were fused by overlap PCR and then cloned into the TRV2‐LIC vector. The CC domain of NbZAR1 (nucleotides 1–420), Pvr4 (nucleotides 1–495) or R3a (nucleotides 1–480) was fused to the eGFP (enhanced green fluorescent protein) epitope by cloning into the pCAMBIA2300‐C‐eGFP‐LIC vector. To produce Cit‐NbPMA3^T955A^ and Cit‐NbPMA3^T955E^, Cit‐NbPMA3 wild type plasmid was amplified with primer sets given in the primer list. To generate autoactive mutants of ANL309 and NbZAR1, a Phusion Site‐Directed Mutagenesis Kit was used (Thermo Fisher). The all constructs were verified by sequencing and introduced into GV3101. The primers used for cloning are listed in Table S1.

### Virus‐induced gene silencing

Virus‐induced gene silencing (VIGS) was performed as described by Liu *et al*. (2002). Briefly, 2‐wk‐old *N. benthamiana* plants were used. *Agrobacterium tumefaciens* strain GV3101 harboring TRV1 and TRV2 and the corresponding fragments of the desired genes were grown overnight at 30°C in LB medium. Harvested cells were resuspended in infiltration buffer (10 mM MES, 10 mM MgCl_2_ and 150 μM acetosyringone, pH 5.6) to a final OD_600_ of 0.3. Resuspended cells were incubated for 1–3 h at room temperature. Cell suspensions of TRV1 and TRV2 were mixed at a 1 : 1 ratio before infiltration. After 2 wk, the upper leaves were used for further experiments. To estimate the efficiency of silencing, gene transcripts were measured by quantitative reverse transcription PCR (qRT‐PCR). Total RNA was isolated from the leaves of silenced plants using TRIzol reagent (Molecular Research Center Inc., Cincinnati, OH, USA), and cDNA was synthesized using SuperScript II Reverse Transcriptase (Invitrogen). qRT‐PCR was performed using a CFX96 Touch Real‐Time PCR Detection System (Bio‐Rad) and SsoAdvanced Universal IT SYBR Green Supermix (Bio‐Rad). *Elongation factor‐1* α (*EF‐1a*) was used as an internal standard. Gene‐specific primers used for expression analysis are listed in Table S1.

### Purification and identification of CC^A^309 protein complex

To identify the putative target complex of CC^A^309 *in planta*, coimmunoprecipitation (co‐IP) was mostly performed as described previously (Win *et al*., 2011). Briefly, *N. benthamiana* leaves infiltrated with *Agrobacterium* cells carrying CC^A^309‐3×FLAG and eGFP‐3×FLAG, respectively, were harvested 28 h post‐infiltration (hpi) and used for purification of the protein complex. To eliminate ribulose‐1,5‐bisphosphate carboxylase/oxygenase from the protein extract, we added 1% protamine sulfate solution (final concentration: 0.06%) to the total protein extract and the mixture was incubated for 30 min on ice (Gupta & Kim, 2015). After centrifugation at 12 000 ***g*** for 10 min at 4°C, the supernatant fraction was mixed with the anti‐FLAG resin. Eluted proteins were run on an SDS‐PAGE gel and the gel was stained with Coomassie blue. Proteins in the whole lane were subjected to MS analysis by the Taplin facility at Harvard University.

### Co‐immunoprecipitation assay

*Agrobacterium*‐mediated transient expression in *N. benthamiana* was performed as described, with some modifications (Mang *et al*., 2017). Briefly, *Agrobacterium* GV3101 (OD_600_ = 0.5) carrying different vectors tagged with 6 × HA, 3 × FLAG, eGFP or 6 × Myc was syringe‐infiltrated into 4‐wk‐old *N. benthamiana* leaves. The infiltrated leaves were collected at 36 hpi for co‐IP. The GFP‐tagged proteins were immunoprecipitated with 10 µl α‐GFP agarose beads (MBL International, Woburn, MA, USA) in 500 µl co‐IP buffer (150 mM NaCl, 50 mM Tris‐HCl, pH 7.5, 2 mM EDTA, 10 mM DTT, 0.2% Triton X‐100 and 1 : 100 complete protease inhibitor cocktail (Roche)). A small aliquot of sample in co‐IP buffer was used as the input control before addition of α‐GFP agarose beads. The co‐IP samples were gently rotated for 3 h at 4°C. The beads were collected and washed more than five times with washing buffer (500 mM NaCl, 25 mM Tris‐HCl, pH 7.5, 1 mM EDTA, and 0.15% NP‐40). Samples were analyzed by immunoblotting with an appropriate antibody.

### Bimolecular fluorescence complementation

The pCAMBIA2300‐LIC vector was modified for cloning the bimolecular fluorescence complementation (BiFC) construct to contain YN (residues 1–155 of yellow fluorescent protein (YFP)) or YC (residues 156 to the stop codon of YFP). CC^A^309 was fused to YN at the C‐terminus in the pCAMBIA2300‐LIC‐YN vector. NbPMA3 or NbPIP2;1 was fused with YC at the C‐terminus in the pCAMBIA2300‐LIC‐YC vector. CC^A^309‐YN was transiently expressed with NbPMA3‐YC or NbPIP2‐YC with p19 silencing suppressor by agroinfiltration at a 1 : 1 : 1 ratio in *N. benthamiana* leaves. Then, leaves were treated with 2 mM LaCl_3_ at 16 hpi to inhibit CC^A^309‐induced cell death. Imaging was performed using a confocal microscope (Leica SP8 X) at 2 d post‐infiltration (dpi). To observe BiFC fluorescence and Chl autofluorescence, a white light laser at excitation wavelengths of 514 and 633 nm, and emission wavelengths of 525–580 and 650–720 nm, was used. Images were processed using Las X software.

### Quantification of intensity of cell death

Cell death was quantified by measuring Chl fluorescence using a closed FluorCam (Photon Systems Instruments (PSI), Drasov, Czech Republic) and FluorCam 7.0 software (PSI). Detached leaves were exposed to a super pulse in a closed chamber, and minimum fluorescence (*F*_0_), maximum fluorescence (*F*_m_) and maximum quantum yield of photosystem II (PSII; *F*_v_/*F*_m_) were determined using the default *F*_v_/*F*_m_ protocol. The *F*_v_/*F*_m_ value for empty vector (EV)‐infiltrated leaves was around 0.77.

### Phylogenetic tree analysis

The amino acid sequences of PMAs in *A. thaliana*, *N. benthamiana* and *Nicotiana plumbaginifolia* were aligned using Muscle (Edgar, 2004). The alignment was then used to construct a phylogenetic tree using the maximum‐likelihood method in Mega7 (Kumar *et al*., 2016). Evolutionary distances were computed using the JTT matrix‐based method with a bootstrap test (500 replicates).

### Fusicoccin treatment

A fusicoccin (1 mM; Sigma‐Aldrich F0537) stock solution was prepared in ethanol and diluted to 1 μM in distilled water. At 12–16 hpi, 1 μM fusicoccin or mock (0.1% ethanol) were infiltrated into the leaf region that had been infiltrated with *Agrobacteria* for transient expression of the indicated genes. To determine whether fusicoccin inhibits R protein‐mediated cell death, *Pto*/*AvrPto* (for activation of Prf), *HRT*/*TCV‐CP*, *Pvr4*/*PepMoV‐NIb*, full‐length *RPS2*, *NbZAR1^D481V^* (the autoactive form of *NbZAR1*), *N*/*p50*, full‐length *SNC1*, full‐length *RPS4*, *R3a*/*Avr3a* and *Rx*/*PVX‐CP* were expressed in *N. benthamiana*, followed by treatment with 1 μM fusicoccin 16 h later.

### Confocal microscopy

Two ratiometric pHluorin sensors, PM‐APO and PM‐CYTO (a gift from Dr Nadine Paris, Université de Montpellier, Montpellier, France), were used to measure the pH on either side of the adjacent PM (Martiniere *et al*., 2018). *Nicotiana benthamiana* leaves were infiltrated with a mixture of *Agrobacteria* harboring a pH sensor and XVE:CC^A^309. Two days after infiltration, 10 μM β‐estradiol solution was sprayed onto the *Agrobacterium*‐infiltrated region to activate CC^A^309 expression. Confocal microscopic observation and quantification of the fluorescence signals was performed as described with modifications. Observations were performed with a Leica SP8 X microscope, using a 20× water objective, with the same WLL laser at 20% of 476 nm and at 20% of 496 nm output. Emission was detected at 505 and 550 nm, with the pinhole set to 1 airy unit. For DiBAC_4_(3) (*bis*‐(1,3‐dibutylbarbituric acid) trimethine oxonol) imaging, leaf disks were incubated in DiBAC_4_(3) (10 μM; Invitrogen B438) for 30 min. For detection, an excitation wavelength of 488 nm was used, and the fluorescence signal emitted between 505 and 560 nm was detected. Calibration curves were obtained *in situ* using proteins purified from *Escherichia coli* and diluted in buffers of various pH. To quantify the intensity of the fluorescence signal, images were analyzed using ImageJ software (https://imagej.nih.gov/ij/). After subtracting background noise, the average mean gray value was calculated for each channel, and ratio images were generated.

### PMA activity assay

Plasma membrane H^+^‐ATPases activity was assayed using an ATPase assay kit (Colorimetric; Abcam, Cambridge, MA, USA), with some modifications. Briefly, microsomal fractions were isolated from *N. benthamiana* leaves transiently overexpressing GFP or CC^A^309‐GFP by homogenization in extraction buffer (250 mM sucrose, 50 mM Hepes‐KOH (pH 7.5), 5% glycerol, 50 mM sodium pyrophosphate decahydrate, 1 mM sodium molybdate dihydrate, 25 mM sodium fluoride, 10 mM EDTA, 0.5% (w/v) polyvinylpyrrolidone, 3 mM DTT, 1 : 100 cOmplete protease inhibitor cocktail (Roche) and 10 nM calyculin A). Total protein extracts were centrifuged at 8000 ***g*** for 10 min. The supernatant was collected and ultracentrifuged at 100 000 ***g*** for 30 min, and the pellet was resuspended in resuspension buffer (5 mM BTP‐MES (pH 7.5), 5 mM KCl, 0.1 mM EDTA, and 6% (w/v) sorbitol). For the ATPase assay, 3 µg of the microsomal fraction was incubated at 30°C for 30 min in 100 µM Na_2_MoO_4_ (an inhibitor of acid phosphatases), 1 mM NaN_3_ (an inhibitor of mitochondrial ATPase) or 50 mM NaNO_3_ (an inhibitor of tonoplast ATPase). To measure basal phosphate levels of sample, the same amount of the microsomal fraction was incubated without ATPase substrate. The amount of phosphate ions was quantified by measuring the absorbance of malachite green at 650 nm according to a standard curve acquired by reacting 0, 1, 2, 3, 4, or 5 nmol phosphate solution with malachite green. The fractions were confirmed by western blot with α‐PMA for the microsomal‐enriched fraction.

### Yeast‐two‐hybrid assay

The Gal4 DNA binding domain‐fused 14‐3‐3 or CC^A^309 was constructed using pGBKT7 (PT3248‐5; Clontech, Palo Alto, CA, USA) and introduced into *Saccharomyces cerevisiae* Y2HGold strain (Clontech) by selection with SD/‐Trp. The Gal4 activation domain‐fused fragments of PMA3 (N, M or C) were constructed using pGADT7 (PT3249‐5; Clontech) and introduced into the Y187 strain by selection with SD/‐Leu. T‐pGADT7 was used as a negative control for the interaction. Cotransformants containing pGBKT7 and pGADT7 were selected on plates lacking leucine and tryptophan (DDO). Successfully transformed yeast cells were suspended in water, and 10 µl droplets of 10‐fold serial dilutions were spotted onto DDO or SD/‐Leu/‐Trp/‐His (TDO) plates containing 100 ng ml^−1^ Aureobasidin A (TDO/A). The plates were incubated at 30°C for 4 d (DDO) or 6 d (TDO/A).

### Pathogen assay

Bacterial cultures were centrifuged and resuspended in 10 mM MgCl_2_. To assess bacterial viability, 4‐wk‐old *Arabidopsis* plants were infiltrated with a bacterial inoculum of OD_600_ = 0.1 (*Pst* carrying *AvrRpt2*) or 0.2 (*Pst*‐D36E carrying *PopP2*). FC (1 μM) was infiltrated into the bacteria‐infiltrated area 3 h after inoculation. Leaf disks were collected at 7 hpi and ground in sterile water. To measure bacterial growth, *Pst* carrying *AvrRpt2* was infiltrated into Col‐0 or *rps2*. FC was applied as described above. Leaf disks were harvested 1 h (Day 0) or 3 d (Day 3) after FC infiltration. The bacterial titer was determined by plating and serial dilution.

### Statistical analysis

Graphs were generated by Prism 7 (GraphPad, La Jolla, CA, USA). The figure legends state the type of statistical test used. Error bars represent the SD or SE of the mean.

## Results

### PMAs interact with CC^A^309 and negatively regulate CC^A^309‐mediated cell death

To gain insight into the mechanism underlying NLR‐mediated cell death, we investigated the primary target(s) of the NLR immune complex. During activation of NLRs after recognition of the cognate effector, various proteins involved in protein stabilization and effector perception associate with the NLR complex (Liu *et al*., 2004; van der Hoorn & Kamoun, 2008). Moreover, the complicated regulatory mode of NLRs imposes constraints on cell death studies. We therefore used CC^A^309, an autoactive CC domain of ANL309, to identify novel immune components involved in the NLR‐mediated cell death process. To identify components of the CC^A^309 complex, the protein complex extracted from *N. benthamiana* leaves expressing *CC^A^309‐3×FLAG* was analyzed by HPLC‐MS/MS. The Sequest algorithm was used to search all MS/MS spectra against the *N. benthamiana* genome (Eng *et al*., 1994) (Table S2). To validate the interactor proteins identified from MS, several candidates showing high peptide scoring were individually subjected to a co‐IP assay with CC^A^309 (Table S2). Among the interacting candidates, NbPMA3 was selected for further study since PMA is essential for plant growth and function to establish the electrochemical gradient at the PM, where CC^A^309 targets (Lee *et al*., 2021) (Fig. S2).

**Fig. 1:**
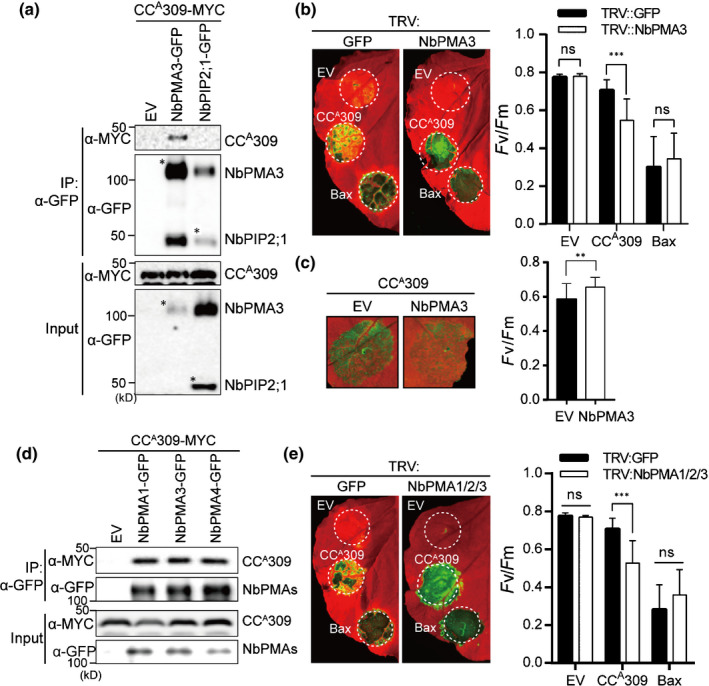
CC^A^309 is associated with NbPMAs, which negatively regulate CC^A^309‐induced cell death. (a) CC^A^309 associates with NbPMA3. *CC^A^309‐MYC* was coexpressed with empty vector (EV), *NbPMA3‐GFP* or *NbPIP2;1‐GFP* in an *Agrobacterium*‐mediated transient assay using 4‐wk‐old *Nicotiana benthamiana*. NbPIP2;1, which is localized in the plasma membrane, was used as a negative control. The leaves were collected at 2 d post‐infiltration (dpi) and β‐estradiol was treated at 1.5 dpi for induction of *CC^A^309‐MYC* expression. Protein extracts were immunoprecipitated with an anti‐GFP antibody (IP:α‐GFP) and immunoblotted with α‐GFP or α‐MYC (upper two panels). Protein inputs are shown with immunoblotting before IP (lower two panels). Asterisks indicate the expected sizes of proteins. (b) Silencing of *NbPMA3* enhances CC^A^309‐induced cell death in *N*. *benthamiana*. Plants silenced by *TRV:GFP* or *TRV:NbPMA3* were agroinfiltrated with EV, *CC^A^309‐FLAG* or *Bax* 2 wk after silencing. The leaves were photographed at 3 dpi (left panel); the degree of cell death was assessed by quantification of quantum yield of photosystem II (*F*_v_/*F*_m_) (right panel). Human Bax and EV were used as a positive or negative control for cell death, respectively. The white dashed circles indicate agroinfiltrated areas. The experiment was performed three times with at least four plants for each TRV construct. Data are presented as the mean ± SD (*n* = 12). Significance was determined using a *t*‐test, with asterisks denoting statistically significant differences. ***, *P* < 0.001; ns, not significant. (c) Overexpression of *NbPMA3* compromises the CC^A^309‐induced cell death in *N*. *benthamiana*. *CC^A^309‐MYC* was coexpressed with EV or *NbPMA3‐GFP* in 4‐wk‐old *N*. *benthamiana* plants. Cell death was photographed at 3 dpi (left panel) and quantified as *F*_v_/*F*_m_ (right panel). Data are represented as mean ± SD (*n* = 18). Asterisks denote a significant difference (*t*‐test; **, *P* < 0.01). (d) CC^A^309 interacts with multiple *NbPMAs in planta*. *CC^A^309‐MYC* was coexpressed with EV, *NbPMA1‐*, *NbPMA3‐* or *PMA4‐GFP* in 4‐wk‐old *N*. *benthamiana* plants. Protein extracts were immunoprecipitated with an anti‐GFP antibody (IP:α‐GFP) and immunoblotted with α‐GFP or α‐MYC (upper two panels). Protein inputs and immunoblotting before immunoprecipitation are shown (lower two panels). (e) Silencing of *NbPMAs* enhances CC^A^309‐induced cell death in *N*. *benthamiana*. *Agrobacterium* carrying *CC^A^309‐FLAG*, or *Bax* was infiltrated into *NbPMAs*‐silenced plants at 2 wk after virus‐induced gene silencing. The leaves were photographed at 3 dpi (left panel), and cell death was quantified as quantum yield, *F*_v_/*F*_m_ (right panel). Data are presented as the mean ± SD (*n* = 9). Asterisks indicate a significant difference with *TRV:GFP* as determined by Student’s *t*‐test (***, *P* < 0.001; ns, not significant). All experiments were repeated three times, each with similar results.

To further demonstrate the *in vivo* association of NbPMA3 with CC^A^309, we performed co‐IP and BiFC assays. NbPIP2, a homolog of the abundant PM‐localized AtPIP2;1 from *Arabidopsis* (Byrt *et al*., 2017), was used as a negative control. The results indicate that NbPMA3 associates with CC^A^309 but not with NbPIP2 (Figs 1a, S3a). Despite the fact that NbPIP2 colocalized with CC^A^309 at the PM (Fig. S3b), the YFP signal was only detected on the PM of cells coexpressing *NbPMA3‐YC* and *CC^A^309‐YN*, but not in the cells coexpressing *NbPIP2‐YC* and *CC^A^309‐YN* (Fig. S3a). Together, these results indicate that CC^A^309 associates specifically with NbPMA3 *in planta*.

To determine whether the association between NbPMA3 and CC^A^309 is functionally implicated in CC^A^309‐induced cell death, we silenced *NbPMA3* in *N. benthamiana* using VIGS; silencing efficiency was confirmed by qRT‐PCR (Fig. S4a). Next, CC^A^309 was expressed transiently in *NbPMA3*‐silenced plants. Human BCL2‐associated X (Bax), a mitochondrial apoptosis regulator, was used as a control (Xing *et al*., 1996). Interestingly, CC^A^309‐induced cell death, but not Bax‐induced cell death, was enhanced significantly in *NbPMA3*‐silenced plants compared with that in *GFP*‐silenced plants (Fig. 1b, left panel). By contrast, CC^A^309‐induced cell death in *N. benthamiana* was compromised by overexpression of *PMA3* (Fig. 1c, left panel). The degree of cell death was inferred from measurement of quantum yield of PSII (*F*_v_/*F*_m_) (Fig. 1b,c, right panel). Expression of protein was measured by western blotting (Fig. S4b,c), showing that the abundance of CC^A^309 was slightly increased in *PMA3*‐silenced plants. We cannot exclude the possibility that enhanced CC^A^309‐induced cell death in *PMA3*‐silenced plants is attributed to stabilization of CC^A^309 protein. These results imply that PMA3 plays a critical role in CC^A^309‐induced cell death.

Plants possess multiple, functionally redundant isoforms of differentially expressed PMAs (Haruta *et al*., 2010). We identified additional putative PMAs from the *N. benthamiana* genome (Bombarely *et al*., 2012), and constructed phylogenic trees including PMAs of *N. plumbaginifolia* and *A. thaliana* (Fig. S5a). Regardless of the phylogenetic clade, co‐IP assays revealed that CC^A^309 also associated with NbPMA1 and NbPMA4, suggesting that CC^A^309 interacts with multiple PMAs *in planta* (Fig. 1d). Consistent with these results, silencing of multiple *PMAs* (*PMA1*/*2*/3) enhanced CC^A^309‐induced cell death significantly (Fig. 1e). Silencing efficiency and protein expression were quantified by qRT‐PCR and immunoblot analyses, respectively (Fig. S4d,e). Although the abundance of CC^A^309 was decreased in *PMA1*/*2*/3‐silenced plants, CC^A^309‐mediated cell death was enhanced. These results suggest that enhanced cell death induced by CC^A^309 *PMA1*/*2*/3‐silenced plants does not result from an increased abundance of CC^A^309 (Fig. S4e). *PMA1*/*2*/*3*/*4*‐silenced plants were also generated but not subjected to the test due to extremely abnormal growth (Fig. S5b). Overall, we conclude that CC^A^309 associates with multiple, functionally redundant PMAs in *N. benthamiana* to trigger cell death.

### CC^A^309 inhibits PMA activity, resulting in alkalization of the apoplast and PM depolarization

**Fig. 2:**
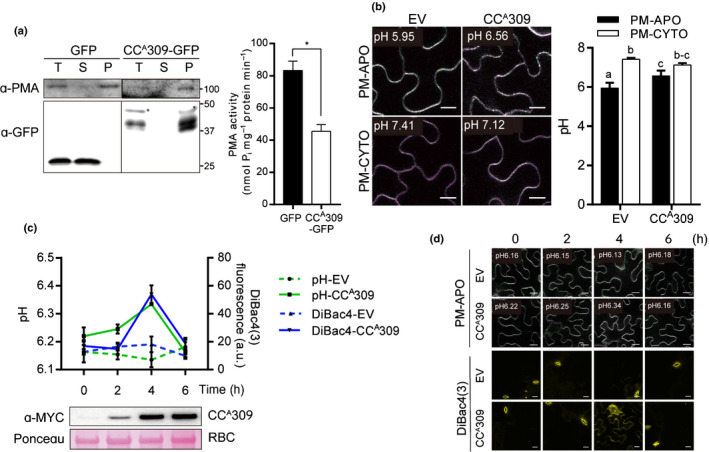
CC^A^309 inhibits PMA activity, resulting in alkalization of apoplast and plasma membrane depolarization. (a) Decreased PMA activity by CC^A^309 in *Nicotiana benthamiana*. Total protein extracted from GFP‐ or CC^A^309‐expressed cells was separated into soluble (S) and microsomal (M) fractions and PMA activity was measured in the microsomal fraction. The fractions were confirmed by western blot with α‐PMA for the microsomal‐enriched fraction (left panel). Asterisks indicate the expected sizes of proteins. Plasma membrane (PM) proteins were purified from GFP‐ or CC^A^309‐GFP‐expressing leaves of *N*. *benthamiana*. The PMA activity of CC^A^309‐GFP‐expressing leaves was significantly decreased compared to GFP‐expressing leaves (right panel). Experiments were repeated three times; results from one representative experiment are shown as mean ± SD. An asterisk indicates a significant difference (paired *t*‐test; *, *P* < 0.05). (b) Elevated apoplastic pH and decreased cytoplasmic pH during CC^A^309‐induced cell death in leaf epidermal cells of *N*. *benthamiana*. *Agrobacterium* harboring β‐estradiol inducible *XVE:CC^A^309* or EV were coinfiltrated with apoplastic (PM‐APO) or cytoplasmic (PM‐CYTO) pH sensors in *N*. *benthamiana*. β‐Estradiol was treated at 24 h post‐infiltration (hpi). Pictures were taken by using the same setting for the confocal microscope at 1 h after β‐estradiol treatment (left panel); the mean pH value is shown on the top of each image. Pseudo color is indicative of fluorescence intensity. Bars, 15 μm. The pH values were calculated using *in vitro* calibration with a recombinant pHluorin (see Supporting Information Fig. S5 and the Materials and Methods section). The data are presented as the mean ± SD (*n* = 6). Different letters indicate statistically significant differences (one‐way ANOVA followed by Sidak’s test (*P* < 0.05)). (c) CC^A^309‐induced apoplastic alkalization and depolarization of the plasma membrane. For measuring apoplastic pH, *Agrobacterium* harboring β‐estradiol inducible *XVE:CC^A^309* or EV were coinfiltrated with apoplastic pH sensors (PM‐APO) in *N*. *benthamiana*. β‐Estradiol was treated at 24 hpi. For detection of depolarized cells, *CC^A^309*‐ or EV‐expressing cells were stained with DiBAC_4_(3). Images were taken by confocal microscopy and the intensity of DiBAC_4_(3) fluorescence was quantified. All of the images were quantified using imageJ software. Protein expression was confirmed by immunoblotting with an α‐MYC antibody and Rubisco (RBC) was stained with Ponceau S to assess protein loading control (lower panel). Data are presented as the mean ± SE (*n* ≥ 8). (d) Representative images obtained by confocal microscopy at each time point in (c). Bars, 20 μm. All experiments were repeated three times with similar results.

To understand how CC^A^309 triggers HR‐associated cell death via PMAs, the molecular mechanism underlying the regulation of PMA was examined in detail. First, we determined whether CC^A^309 affects the activity of PMAs *in vivo*. An enriched plasma membrane fraction was obtained from *N. benthamiana* leaves expressing GFP and CC^A^309, respectively, and fractionation was confirmed by immunoblot with α‐PMA (Fig. 2a, left panel). A purified microsomal fraction was used for determination of ATPase activity. Proton pump activity was assayed spectrophotometrically by measuring the inorganic phosphate level. Interestingly, we detected a significantly low level of PMA activity by transient overexpression of CC^A^309 compared with GFP in *N. benthamiana* (Fig. 2a, right panel).

Next, we monitored the pH change in both cellular compartments during CC^A^309‐induced cell death, because PMAs actively pump protons out of the cytoplasm into the apoplast. To measure the pH in the cytoplasm and apoplast, we utilized the ratiometric pHluorin sensors PM‐CYTO and PM‐APO, respectively (Martiniere *et al*., 2018). *Agrobacterium tumefaciens* carrying *CC^A^309* and *PM‐CYTO* or *PM‐APO* were infiltrated into *N. benthamiana* leaves, and β‐estradiol was used to initiate expression of CC^A^309 at 24 h post‐infiltration. The fluorescence ratio was assessed in epidermal cells, and the corresponding pH values were calculated using *in vitro* calibration with recombinant pHluorin (Fig. S6). Compared with cells expressing the EV control, the apoplastic pH was higher (by *c*. 0.6), whereas the cytoplasmic pH was lower (by *c*. 0.3), in *CC^A^309*‐expressing cells (Fig. 2b). Interestingly, apoplastic alkalization by CC^A^309 was diminished in cells coexpressing CC^A^309 and *NbPMA3* (Fig. S7), which is consistent with the compromised cell death observed in *N. benthamiana* leaves coexpressing *NbPMA3* and *CC^A^309* (Fig. 1c). Together, these findings imply that CC^A^309 triggers the inhibition of proton pump activity and apoplastic alkalization, and that this alkalization is reversible by overexpression of PMAs.

Plasma membrane H^+^‐ATPases are responsible for maintaining an acidic environment in the apoplast and creating an electrochemical potential across the PM. This electrochemical potential is coupled with regulation of the activity of most transporters and ion channels in the PM (Haruta *et al*., 2015). We speculated that the disturbance of PM potential is accompanied by CC^A^309‐induced cell death. Therefore, we monitored changes in PM potential and apoplastic pH simultaneously during CC^A^309‐induced cell death in *N. benthamiana* leaves. To assess the PM potential, we utilized the voltage‐sensitive fluorescent dye, DiBAC_4_(3), which exhibits enhanced fluorescence when bound to depolarized membranes (Konrad & Hedrich, 2008). While apoplastic pH was gradually increased until 4 h postinduction, PM‐depolarized cells were particularly observed in 4 h after induction (Fig. 2c,d). Together, these results indicate that CC^A^309 triggers alkalization of the extracellular space, followed by depolarization of the PM.

### CC^A^309 inhibits the 14‐3‐3‐mediated activation of PMA

**Fig. 3:**
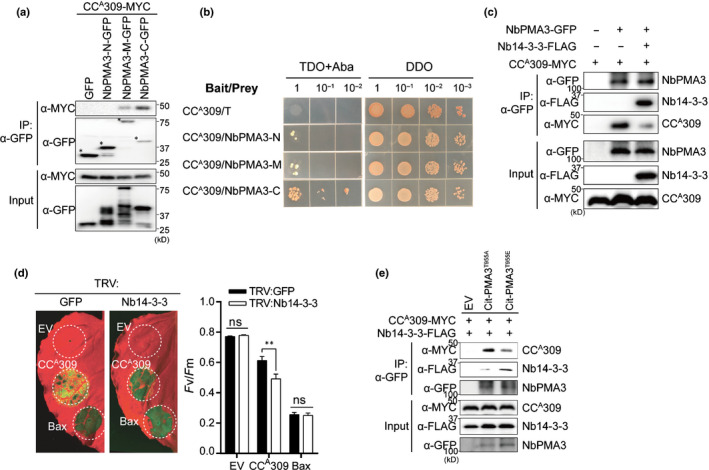
CC^A^309 suppresses PMA activity by interacting with the inactive form of PMA. (a) CC^A^309 interacts with the cytosolic central domain (NbPMA3‐M) and the C‐terminal domain (NbPMA3‐C), but not with the N‐terminal domain (NbPMA3‐N) of NbPMA3 *in planta*. *CC^A^309‐MYC* was coexpressed with EV, *NbPMA3‐N‐GFP*, *NbPMA3‐M‐GFP* or *NbPMA3‐C‐GFP* in *Nicotiana benthamiana*. Protein extracts were immunoprecipitated with α‐GFP (IP:α‐GFP) and immunoblotted with α‐MYC or α‐GFP (upper two panels). Protein inputs and immunoblots before IP are shown (lower two panels). (b) CC^A^309 interacts with a C‐terminal domain (NbPMA3‐C), but not an N‐terminal domain (NbPMA3‐N) and a cytosolic central domain (NbPMA3‐M) of NbPMA3 in yeast. Gal4 DNA binding domain‐fused CC^A^309 was introduced into Y2HGold, and the Gal4 activation domain‐fused three cytosolic domains used in (a) were introduced into Y187, respectively. T was used as a negative control for interaction. The transformed yeast cells (serially diluted 10‐fold) were spotted onto DDO (‐L, ‐T) and TDO (‐L, ‐T, ‐H) containing Aureobasidin A (AbA) plates. (c) Decreased interaction between NbPMA3 and CC^A^309 by Nb14‐3‐3. NbPMA3‐GFP and/or CC^A^309‐MYC and/or EV were coexpressed with or without Nb14‐3‐3‐FLAG in *N*. *benthamiana*. Co‐IP was carried out with α‐GFP and analyzed by western blot with α‐GFP, α‐FLAG or α‐MYC antibodies (upper three panels). Protein inputs and immunoblots before IP are shown (lower three panels). (d) Silencing of *Nb14‐3‐3* enhances CC^A^309‐induced cell death in *N*. *benthamiana*. Plants silenced by *TRV:GFP* or *TRV:Nb14‐3‐3* were agroinfiltrated with EV, *CC^A^309‐FLAG* or *Bax* 3 wk after silencing. Leaves were photographed at 3 d post‐infiltration (dpi) (left panel) and the degree of cell death was assessed by quantification of the quantum yield (*F*_v_/*F*_m_) (right panel). Human Bax and EV were used as positive or negative controls, respectively, for cell death. The white dashed circles indicate agro‐infiltrated areas. Significance was determined using a *t*‐test, with asterisks denoting statistically significant differences. **, *P* < 0.01; ns, not significant. (e) CC^A^309 strongly interacts with null‐phospho NbPMA3 (Citrine (Cit)‐PMA3^T955A^) than with the phospho‐mimic NbPMA3 (Cit‐PMA3^T955E^) mutants. CC^A^309‐MYC and Nb14‐3‐3‐FLAG were coexpressed with EV, Cit‐PMA3^T955A^ or Cit‐PMA3^T955E^ in *N*. *benthamiana*. Co‐IP was carried out with an α‐GFP and analyzed by western blot with α‐GFP, α‐FLAG or α‐MYC antibodies (upper three panels). Protein inputs and immunoblots before IP are shown (lower three panels). All experiments were repeated three times with similar results.

Plasma membrane H^+^‐ATPases contain three major cytosolic domains, including a central catalytic domain and a C‐terminal autoinhibitory domain. PMA activity is generally regulated by posttranslational modifications, especially the phosphorylation of various serine and threonine residues in the C‐terminal regulatory domain (Haruta *et al*., 2015). To determine which domain(s) of NbPMA3 interact with CC^A^309, we performed co‐IP and yeast two‐hybrid assays to investigate the association of CC^A^309 with three cytosolic domains of NbPMA3, NbPMA3‐N (1–64 amino acids (aa)), NbPMA3‐M (305–650 aa), and NbPMA3‐C (846–956 aa). Although CC^A^309 interacted with both the central domain (NbPMA3‐M) and C‐terminal domain (NbPMA3‐C) of NbPMA3, the co‐IP signal was repeatedly stronger with the C‐terminal domain than with the central domain (Fig. 3a). The results of yeast two‐hybrid analyses (Fig. 3b) were similar to those of the co‐IP assay, suggesting that CC^A^309 probably regulates the autoinhibited state of PMAs through association with the C‐terminal regulatory domain.

Plasma membrane H^+^‐ATPases are activated by phosphorylation at the penultimate threonine residue, as it allows for binding of the activator protein 14‐3‐3, thereby relieving the autoinhibition state (Jelich‐Ottmann *et al*., 2001; Elmore & Coaker, 2011). To examine whether CC^A^309 is involved in regulating PMA via the 14‐3‐3‐mediated activation process, we identified the *N. benthamiana* homolog of the *N. plumbaginifolia* 14‐3‐3c protein, which is known to interact with *N. plumbaginifolia* PMA2, and confirmed the association between Nb14‐3‐3 and NbPMA3 by co‐IP (Fig. S8) (Maudoux *et al*., 2000).

Alkalization of the apoplast in *CC^A^309*‐expressing cells implies a dynamic interaction between PMA3 and Nb14‐3‐3 or CC^A^309. We found that coexpression of *Nb14‐3‐3* and *CC^A^309* compromised the association between CC^A^309 and NbPMA3 in co‐IP experiments (Fig. 3c). Therefore, we further investigated the roles of Nb14‐3‐3 in CC^A^309‐induced cell death. To demonstrate the biological significance of 14‐3‐3 in CC^A^309‐induced cell death, we silenced the *Nb14‐3‐3* gene in *N. benthamiana* using VIGS (Fig. 3d); the efficiency of VIGS was confirmed by qRT‐PCR (Fig. S9a). Consistent with the co‐IP data, cell death induced by CC^A^309 in *Nb14‐3‐3*‐silenced plants was significantly greater than that in *GFP*‐silenced plants (Fig. 3d). Protein expression was confirmed by immunoblotting (Fig. S9b). Lastly, because PMA activation relies on phosphorylation of the penultimate threonine residue, we tested whether the activation status of NbPMA3 affects its interaction with CC^A^309. To perform this experiment, we constructed plasmids expressing phosphomimic (T955E) and null‐phospho (T955A) variants of NbPMA3. Co‐IP assays using these variants showed that CC^A^309 interacted more strongly with T955A than with T955E, whereas Nb14‐3‐3 showed the opposite (Fig. 3e). These results suggest that CC^A^309 preoccupies inactive PMA and prevents its association with the activator, Nb14‐3‐3, in plants.

### PM‐localized CNL‐induced cell death is compromised by a PMA activator, fusicoccin

**Fig. 4:**
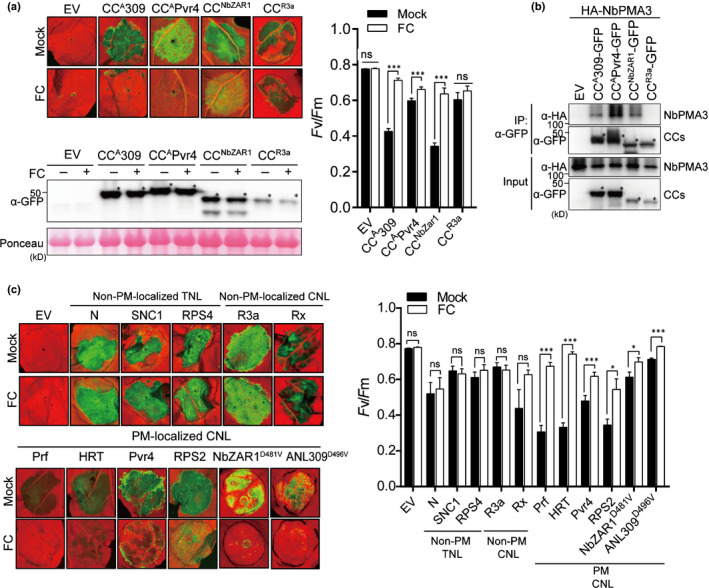
Cell death induced by plasma membrane (PM)‐localized CNLs is inhibited by a PMA activator. (a) Fusicoccin (FC) inhibited the cell death induced by the CC domain of CaANL309, CaPvr4 and NbZAR1, but not that of SdR3a. Agrobacteria carrying the indicated constructs were infiltrated into 4‐wk‐old *Nicotiana benthamiana,* followed by infiltration of 1 μM FC at 16 h post‐infiltration (hpi). The leaves were photographed at 3 d post‐infiltration (dpi) (left upper panel). The proteins were detected by immunoblotting with α‐GFP. Ponceau S staining of Rubisco (RBC) was used as a protein loading control (left lower panel). Asterisks indicate the expected sizes of proteins (left lower panel). The degree of cell death was quantified by calculating the quantum yield (*F*_v_/*F*_m_) (right panel). Data are presented as the mean ± SE (*n* = 5–25). All experiments were performed in triplicate and independent. The significance of the differences was determined using a *t*‐test. ***, *P* < 0.001. (b) The CC domains affected by FC only interact with NbPMA3. *HA‐NbPMA3* was coexpressed with EV or with each CC domain in *N*. *benthamiana*. Protein extracts were immunoprecipitated with α‐GFP (IP:α‐GFP), and immunoblotted with α‐HA and α‐GFP (upper two panels). Protein inputs and immunoblots before IP are shown (lower two panels). Asterisks indicate the expected protein bands. (c) FC inhibits only cell death mediated by PM‐localized full‐length CNL(CC‐NLR)‐type R proteins, but not TNL‐ (TIR‐NLR) or other organelle‐targeting CNLs in *N*. *benthamiana*. Leaves were treated with FC as described in (a). To induce cell death, R genes were expressed with their cognate Avr genes: *Arabidopsis* HRT/turnip crinkle virus coat protein (TCV‐CP), pepper (*Capsicum annuum*) Pvr4/pepper mottle virus RNA‐dependent RNA polymerase (PepMoV‐NIb), tobacco (*Nicotiana tabacum*) N/helicase domain (p50) of the tobacco mosaic virus replicase, potato (*Solanum tuberosum*) R3a/Avr3a, and potato (*Solanum tuberosum*) Rx/potato virus X coat protein, respectively. *Pst* AvrPto + Pto (from *Lycopersicon esculentum*) were used to trigger hypersensitive response cell death mediated by the endogenous NLR protein, Prf in *N. benthamiana* Autoactive R genes (Arabidopsis *SNC1*, *RPS5* or *RPS2*) or autoactive NLR mutants (*C*. *annuum* ANL309^D496V^ or NbZAR1^D481V^) were expressed alone. Images were taken at 3 dpi (left panel), and cell death was quantified by measuring quantum yield (*F*_v_/*F*_m_) (right panel). Data are presented as the mean ± SE (*n* = 5–25). Significance was determined using a *t*‐test. *, *P* < 0.05; ***, *P* < 0.001; ns, not significant. All experiments were performed in triplicate.

To determine whether PMA activity indeed affects CC domain‐induced cell death, we performed additional pharmacological experiments using FC, which irreversibly activates PMA by preventing dissociation of the PMA/14‐3‐3 complex (Baunsgaard *et al*., 1998). FC was applied to *N. benthamiana* leaves expressing different autoactive CC domains, including *CC^A^309*, potyvirus resistance protein in pepper (Pvr4; CC^A^Pvr4), *Xanthomonas campestris* resistance protein in *N. benthamiana* (NbZAR1; CC^NbZar1^) and *Phytophthora infestans* resistance protein in potato (R3a; CC^R3a^), at 16 h postagroinfiltration (Armstrong *et al*., 2005; Kim *et al*., 2018; Schultink *et al*., 2019; Lee *et al*., 2021), and the degree of cell death was assessed at 3 d postinfiltration. Surprisingly, FC only inhibited cell death induced by PM‐localized CC domains such as CC^A^309, CC^A^Pvr4 and CC^NbZAR1^; it did not affect cell death induced by cytosol‐ or endoplasmic reticulum‐localized CC^R3a^ (Fig. 4a, left top and right panel), without critical defects in protein expression (Fig. 4a, left bottom panel). Moreover, co‐IP assays showed that the CC domains of CC^A^309, Pvr4 and NbZAR1, but not that of R3a, interacted with NbPMA3 *in planta* (Fig. 4b). Together, these results suggest that the CC domains of PM‐localized ANL309, Pvr4 and NbZAR1 induce cell death by inhibiting the activity of PMAs, and that suppression of cell death induced by the CC domains of these PM‐localized proteins cab probably be attributed to FC‐induced constitutive activation of PMAs.

To extend our understanding of the role of PMAs in R protein‐mediated cell death, functionally characterized NLRs were subjected to the FC test. Full‐length wild‐type or autoactive mutant *NLRs* were expressed with or without the cognate effector in *N. benthamiana* leaves, which were then subjected to FC treatment. Cell death mediated by PM‐localized CNLs (Prf, HRT, Pvr4, RPS2 and NbZAR1^D481V^), but not that mediated by non‐PM‐localized CNLs (R3a and Rx) and non‐PM‐localized TNLs (N, SNC1 and RPS4), was compromised by FC (Fig. 4c) (Hamel *et al*., 2016; Kim *et al*., 2018; Harant *et al*., 2020). Moreover, we found that full‐length Pvr4 and NbZAR1 interacted with PMA3 in co‐IP assays (Fig. S10a,b). These results suggest that the PMAs control cell death via autoactive CC domain as well as autoactive full‐length NLR and R‐AVR protein interactions.

### PMA activator inhibits the cell death and resistance mediated by PM‐localized CNL, RPS2

**Fig. 5:**
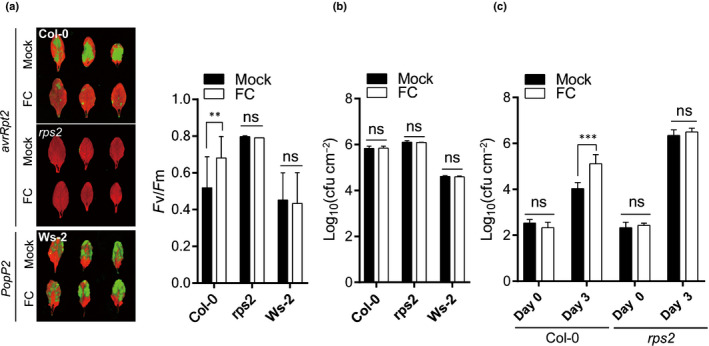
The plasma membrane H^+^‐ATPase (PMA) activator FC compromises the *RPS2*‐mediated hypersensitive response (HR) cell death and disease resistance in *Arabidopsis*. (a) Inhibition of RPS2‐mediated HR cell death by FC in *Arabidopsis*. Four‐week‐old wild‐type Col‐0 and *rps2* mutant were inoculated with *Pst‐avrRpt2* at OD_600_ = 0.1. After 3 h post‐infiltration (hpi), 1 µM FC was infiltrated in *Arabidopsis*. Representative images (left panel) and quantified as *F*_v_/*F*_m_ (right panel) after 6 h. *Pst‐PopP2* with Ws‐2 was used as the TNL‐type, indicating no effect of FC. Data are represented as mean ± SE (*n* = *c*. 3–20). Significance was determined using a *t*‐test. **, *P* < 0.01. (b) FC does not affect bacterial viability. Pathogen‐inoculated leaf samples were collected at 7 hpi. The data are shown as means ± SD (*n* = *c*. 3–6). Significance was determined using a *t*‐test (ns, no significant). (c) RPS2‐mediated immunity is suppressed by FC in *Arabidopsis*. Four‐week‐old wild‐type Col‐0 and *rps2* mutant plants were infiltrated with *Pst‐avrRpt2* at OD_600_ = 0.001. The number of bacteria per area of leaf is represented on a log_10_ scale for day 0 (1 h after FC infiltration) and day 3. Data are presented as mean ± SE (*n* = 5). Significance was determined using a *t*‐test. ***, *P* < 0.001. The above experiments were repeated at least three times with similar results.

Lastly, we reconstituted NLR‐mediated HR in the *Arabidopsis* system. *Arabidopsis* CNL protein RPS2 initiates HR upon recognition of avrRpt2 from *Pseudomonas syringae* pv. *tomato* DC3000 (*Pst*) in the ecotype Col‐0 (Axtell & Staskawicz, 2003). PopP2, a *Ralstonia solanacearum* effector, is perceived by paired TNL proteins RRS1/RPS4 to trigger a strong HR in Ws‐2 (Saucet *et al*., 2015). *Pst* carrying *avrRpt2* (*Pst avrRpt2*) were inoculated into Col‐0, whereas an *rps2* mutant and *Pst‐D36E*, a strain lacking all known effectors, carrying PopP2 (*Pst* PopP2), were inoculated into Ws‐2. As expected, FC treatment reduced *Pst avrRpt2*‐induced cell death significantly in Col‐0. By contrast, FC did not affect *Pst* PopP2‐induced cell death in Ws‐2 (Fig. 5a). FC treatment did not affect the viability of cells harboring *Pst* (Fig. 5b), indicating that FC‐inhibited cell death in plants is not due to the toxic effects of FC on *Pst* proliferation. Surprisingly, we observed significantly enhanced growth of *Pst avrtRpt2* cells in FC‐treated plants compared with mock treatment (Fig. 5c). This result indicates that activation of PMAs not only inhibits cell death induced by the R–AVR gene interaction, but also confers susceptibility to *Pst avrRpt2*. Together, these results indicate that PMA is a pivotal regulator of PM‐associated NLR‐mediated cell death and resistance in plants.

## Discussion

Plasma membrane H^+^‐ATPases are the primary pump that establishes an electrochemical gradient across the PM; as such, they are essential for the survival of all living organisms (Kuhlbrandt, 2004). By pumping protons from the cytosol into the apoplast, PMAs contribute to maintenance of the intracellular and extracellular pH and facilitate the activities of secondary transporters or ion channels. Thus, the activity of PMAs is crucial for plant growth and development under different environmental conditions (Sondergaard *et al*., 2004). Given the importance of PMAs in regulating the basic aspects of plant cell physiology and nutrient transport, pathogens have evolved strategies to manipulate PMA activity during infection (Elmore & Coaker, 2011). Some fungal pathogens secrete toxins that target PMA activity to affect stomata aperture and trigger cell death (Atkinson & Baker, 1989). Elicitors secreted by bacterial pathogens inhibit or activate PMA, which leads to depolarization or hyperpolarization of the PM, respectively (Atkinson & Baker, 1989; Vera‐Estrella *et al*., 1994).

In addition, PMAs appear to initiate defense responses during plant–pathogen interactions mediated by R proteins, especially pattern recognition receptors (PRRs) (Atkinson & Baker, 1989; Vera‐Estrella *et al*., 1994; Xing *et al*., 1996; Liu *et al*., 2009). In *Arabidopsis*, treatment with pathogen‐associated molecular patterns (PAMPs) such as flg22 and lipopolysaccharide triggers stomatal closure to restrict bacterial invasion, which is initiated upon depolarization of guard cells by inhibiting PMAs (Liu *et al*., 2009). However, *ost2‐1D* and *ost2‐2D*, which possess dominant activation mutations in PMA and AHA1, do not respond to PAMP‐mediated stomatal closure, suggesting that AHA1 engages in PRR‐mediated resistance (Liu *et al*., 2009). In tomato, activation of host PMAs by elicitors secreted by incompatible races of *C. fulvum* appears to be mediated by a heterotrimeric G protein, which activates a membrane‐bound phosphatase to induce dephosphorylation of PMAs (Xing *et al*., 1996, 1997). However, involvement of PMAs in NLR‐mediated immunity has not yet been investigated. Interestingly, our observation that the cell death activity of PM‐associated NLRs, including Prf, HRT, Pvr4, RPS2, ANL309 and ZAR1, affected by PMA activity (Fig. 4c) is in line with a previous report showing that PRR FLS2 was found in the same complex with RPS2 (Qi, *et al*., 2011). Thus, these data imply that PRRs and PM‐localized NLRs might initiate defense signaling by regulation of PMA activity.

To date, apoplastic alkalization has mainly been observed during PAMP–PRR interactions. For example, PAMPs such as flg22 or elf18 trigger rapid membrane alkalization and depolarization upon their perception by PRRs such as FLS2 or EFR (Felix *et al*., 1999; Bauer *et al*., 2001; Kunze *et al*., 2004; Jeworutzki *et al*., 2010). Unexpectedly, our experiments using the ratiometric fluorescence sensor PM‐APO revealed PMA‐mediated apoplastic alkalization at the early phase of CC domain‐induced cell death (Fig. 2c,d). Intriguingly, the alkaline status of the extracellular space was prolonged by only 4 h after expression of CC^A^309, and reacidification was subsequently observed in the repeated experiments (Fig. 2c,d). We cannot exclude the possibility that autofluorescence from some dead cells interfered with accurate calculation of the fluorescence signal. Therefore, an alternative pH measurement method was necessary to determine whether reacidification occurred after rapid alkalization of the apoplast.

Pharmacological and biochemical analyses revealed that inhibition of PMA activity underlies PM‐localized CNL‐induced cell death. Interestingly, we showed that NbZAR1 was associated with NbPMA3 (Fig. S10b), and that NbZAR1‐induced cell death was also inhibited by FC treatment (Fig. 4c). Eventually, FC confers disease susceptibility to *Pst avrRpt2* in *Arabidopsis* (Fig. 5c). These results suggest that inhibition of PMA in NLR‐activated cell is required for HR cell death and immune response.

Recent elucidation of the structure of the activated *Arabidopsis* ZAR1 complex revealed that the N‐terminal α‐helix of ZAR1 forms a funnel‐shaped structure, which is required for PM association, HR‐associated cell death and disease resistance (Bi *et al*., 2021). The funnel‐like structure is thought to mediate cell death by forming a pore in the PM. More recently, they revealed that the ZAR1 resistosome forms an ion‐conducting pore and functions as a calcium‐permeable channel with unidentified cations to trigger the plant immune response. Another type of NLR, helper NRG1, also oligomerizes upon activation and translocates to the PM to form a pore‐like calcium channel (Jacob *et al*., 2021). Subsequently, the influx of calcium ions leads to cell death (Jacob *et al*., 2021). Although most experiments demonstrating the association with PMA3 were performed using CC^A^309, rather than full‐length NLR, we found ANL309 has a capacity to self‐association, implying that ANL309 could form a multimeric complex‐like resistosome (Fig. S1). As the ZAR1 resistosome showed a calcium‐permeable channel activity in the absence of any other proteins, further investigation is necessary to determine whether HR cell death requires coordination among PMA‐mediated pH change, depolarization of PM and Ca^2+^ influx via an activated resistosome. Extended research on the role of PMA during inflammasome‐induced cell death in various eukaryotes such as human, mouse and fungal pathogens would provide further insight into the role of the H^+^ pump in innate immunity in plants and other eukaryotic organisms.

Our findings provide new insight into the mechanism by which activated NLRs manipulate host PM component H^+^ pumps in response to pathogenic stimuli. Here, we propose that PMAs act as primary targets of PM‐associated CNLs to facilitate both induction of cell death by disturbing the homeostasis of the PM potential, and via generation of secondary signals, such as Ca^2+^ influx and reactive oxygen species burst, to trigger defense responses and HR‐associated cell death in plants (Fig. S11).

## Author contributions

DC conceived the project; HM, H‐YL, Y‐ES and DC designed the experiments; HM, H‐YL, Y‐ES, JHL, SO, SEL, JK, SJ, HK, HP and SK performed the experiments; HM, H‐YL, Y‐ES and DC wrote the manuscript. H‐YL, Y‐ES and HM contributed equally to this work.

## Supporting information


**Fig. S1** Full‐length ANL309 has a capacity to form homodimers.
**Fig. S2** CC^A^309 is colocalized with NbPMA3 at the plasma membrane.
**Fig. S3** CC^A^309 is associated with NbPMA3, not PM‐localized protein NbPIP2;1.
**Fig. S4** The silencing efficiency and protein expression in *NbPMA3* or *NbPMA1/2/3* silencing plants, and expression of NbPMA3 in CC^A^309 overexpressed plants (Fig. S1).
**Fig. S5** NbPMA3 is a member of the H^+^‐ATPase subfamily that plays essential and redundant functions in plant development.
**Fig. S6** Calibration of the pHluorin response to various external pH buffers.
**Fig. S7** NbPMA3 compromised the CC^A^309‐induced apoplastic alkalization.
**Fig. S8** Nb14‐3‐3 associates with NbPMA3.
**Fig. S9** The silencing efficiency and protein expression in *Nb14‐3‐3* silencing plants.
**Fig. S10** Full‐length R proteins, CaPvr4 and NbZAR1, associate with NbPMA3.
**Fig. S11** Proposed molecular mechanism of plasma membrane‐associated CNL‐mediated cell death.
**Table S1** Primer sequences used in this study.
**Table S2** List of the CC^A^309 interactor candidates identified by MS.
Please note: Wiley Blackwell are not responsible for the content or functionality of any Supporting Information supplied by the authors. Any queries (other than missing material) should be directed to the *New Phytologist* Central Office.

## Data Availability

The data that support the findings of this study are openly available in *bioRxiv* at doi: https://doi.org/10.1101/2020.08.30.274688.

## References

[nph17789-bib-0001] Armstrong MR , Whisson SC , Pritchard L , Bos JI , Venter E , Avrova AO , Rehmany AP , Böhme U , Brooks K , Cherevach I *et al*. 2005. An ancestral oomycete locus contains late blight avirulence gene Avr3a, encoding a protein that is recognized in the host cytoplasm. Proceedings of the National Academy of Sciences, USA 102: 7766–7771. 10.1073/pnas.0500113102 PMC1140420 15894622

[nph17789-bib-0002] Atkinson MM , Baker CJ . 1989. Role of the plasmalemma H^+^‐ATPase in *Pseudomonas syringae*‐induced K/H exchange in suspension‐cultured tobacco cells. Plant Physiology 91: 298–303. 16667014 10.1104/pp.91.1.298 PMC1061990

[nph17789-bib-0003] Axtell MJ , Staskawicz BJ . 2003. Initiation of RPS2‐specified disease resistance in Arabidopsis is coupled to the AvrRpt2‐directed elimination of RIN4. Cell 112: 369–377. 12581526 10.1016/s0092-8674(03)00036-9

[nph17789-bib-0004] Bauer Z , Gomez‐Gomez L , Boller T , Felix G . 2001. Sensitivity of different ecotypes and mutants of *Arabidopsis thaliana* toward the bacterial elicitor flagellin correlates with the presence of receptor‐binding sites. Journal of Biological Chemistry 276: 45669–45676. 11564731 10.1074/jbc.M102390200

[nph17789-bib-0005] Baunsgaard L , Fuglsang AT , Jahn T , Korthout HA , de Boer AH , Palmgren MG . 1998. The 14‐3‐3 proteins associate with the plant plasma membrane H^+^‐ATPase to generate a fusicoccin binding complex and a fusicoccin responsive system. The Plant Journal 13: 661–671. 9681008 10.1046/j.1365-313x.1998.00083.x

[nph17789-bib-0006] Bi G , Su M , Li N , Liang YU , Dang S , Xu J , Hu M , Wang J , Zou M , Deng Y *et al*. 2021. The ZAR1 resistosome is a calcium‐permeable channel triggering plant immune signaling. Cell 184: 3528–3541. 33984278 10.1016/j.cell.2021.05.003

[nph17789-bib-0007] Boller T , Felix G . 2009. A renaissance of elicitors: perception of microbe‐associated molecular patterns and danger signals by pattern‐recognition receptors. Annual Review of Plant Biology 60: 379–406. 10.1146/annurev.arplant.57.032905.105346 19400727

[nph17789-bib-0008] Bombarely A , Rosli HG , Vrebalov J , Moffett P , Mueller LA , Martin GB . 2012. A draft genome sequence of *Nicotiana benthamiana* to enhance molecular plant‐microbe biology research. Molecular Plant–Microbe Interactions 25: 1523–1530. 22876960 10.1094/MPMI-06-12-0148-TA

[nph17789-bib-0009] Byrt CS , Zhao M , Kourghi M , Bose J , Henderson SW , Qiu J , Gilliham M , Schultz C , Schwarz M , Ramesh SA *et al*. 2017. Non‐selective cation channel activity of aquaporin AtPIP2; 1 regulated by Ca^2+^ and pH. Plant, Cell & Environment 40: 802–815. 10.1111/pce.12832 27620834

[nph17789-bib-0011] Edgar RC . 2004. Muscle: multiple sequence alignment with high accuracy and high throughput. Nucleic Acids Research 32: 1792–1797. 15034147 10.1093/nar/gkh340 PMC390337

[nph17789-bib-0012] Elmore JM , Coaker G . 2011. The role of the plasma membrane H^+^‐ATPase in plant‐microbe interactions. Molecular Plant 4: 416–427. 21300757 10.1093/mp/ssq083 PMC3107590

[nph17789-bib-0013] Eng JK , McCormack AL , Yates JR . 1994. An approach to correlate tandem mass spectral data of peptides with amino acid sequences in a protein database. Journal of the American Society for Mass Spectrometry 5: 976–989. 24226387 10.1016/1044-0305(94)80016-2

[nph17789-bib-0014] Felix G , Duran JD , Volko S , Boller T . 1999. Plants have a sensitive perception system for the most conserved domain of bacterial flagellin. The Plant Journal 18: 265–276. 10377992 10.1046/j.1365-313x.1999.00265.x

[nph17789-bib-0055] Gupta R , Kim ST . 2015. Depletion of RuBisCO protein using the protamine sulfate precipitation method. Methods in Molecular Biology 1295: 225–233. 25820725 10.1007/978-1-4939-2550-6_17

[nph17789-bib-0016] Hamel LP , Sekine KT , Wallon T , Sugiwaka Y , Kobayashi K , Moffett P . 2016. The chloroplastic protein THF1 interacts with the coiled‐coil domain of the disease resistance protein N' and regulates light‐dependent cell death. Plant Physiology 171: 658–674. 26951433 10.1104/pp.16.00234 PMC4854715

[nph17789-bib-0017] Harant A , Sakai T , Kamoun S , Adachi H . 2020. A vector system for fast‐forward *in vivo* studies of the ZAR1 resistosome in the model plant *Nicotiana benthamiana* . bioRxiv. doi: 10.1101/2020.05.15.097584. PMC8774824 34633454

[nph17789-bib-0018] Haruta M , Burch HL , Nelson RB , Barrett‐Wilt G , Kline KG , Mohsin SB , Young JC , Otegui MS , Sussman MR . 2010. Molecular characterization of mutant Arabidopsis plants with reduced plasma membrane proton pump activity. Journal of Biological Chemistry 285: 17918–17929. 20348108 10.1074/jbc.M110.101733 PMC2878554

[nph17789-bib-0019] Haruta M , Gray WM , Sussman MR . 2015. Regulation of the plasma membrane proton pump (H^+^‐ATPase) by phosphorylation. Current Opinion in Plant Biology 28: 68–75. 26476298 10.1016/j.pbi.2015.09.005 PMC4679459

[nph17789-bib-0020] van der Hoorn RA , Kamoun S . 2008. From guard to decoy: a new model for perception of plant pathogen effectors. Plant Cell 20: 2009–2017. 18723576 10.1105/tpc.108.060194 PMC2553620

[nph17789-bib-0021] Horsefield S , Burdett H , Zhang XX , Manik MK , Shi Y , Chen J , Qi TC , Gilley J , Lai JS , Rank MX *et al*. 2019. NAD^+^ cleavage activity by animal an plant TIR domains in cell death pathways. Science 365: 793–799. 31439792 10.1126/science.aax1911

[nph17789-bib-0056] Jacob P , Kim NH , Wu F , El‐Kasmi F , Chi Y , Walton WG , Dangl JL . 2021. Plant “helper” immune receptors are Ca^2+^‐permeable nonselective cation channels. Science 373: 3556. 10.1126/science.abg7917 PMC8939002 34140391

[nph17789-bib-0022] Jelich‐Ottmann C , Weiler EW , Oecking C . 2001. Binding of regulatory 14‐3‐3 proteins to the C terminus of the plant plasma membrane H^+^‐ATPase involves part of its autoinhibitory region. Journal of Biological Chemistry 276: 39852–39857. 11517228 10.1074/jbc.M106746200

[nph17789-bib-0023] Jeworutzki E , Roelfsema MRG , Anschutz U , Krol E , Elzenga JTM , Felix G , Boller T , Hedrich R , Becker D . 2010. Early signaling through the Arabidopsis pattern recognition receptors FLS2 and EFR involves Ca^2+^‐associated opening of plasma membrane anion channels. The Plant Journal 62: 367–378. 20113440 10.1111/j.1365-313X.2010.04155.x

[nph17789-bib-0024] Jones JD , Vance RE , Dangl JL . 2016. Intracellular innate immune surveillance devices in plants and animals. Science 354: 1117. 10.1126/science.aaf6395 27934708

[nph17789-bib-0025] Kim S‐B , Lee H‐Y , Choi E‐H , Park E , Kim J‐H , Moon K‐B , Kim H‐S , Choi D . 2018. The coiled‐coil and leucine‐rich repeat domain of the potyvirus resistance protein Pvr4 has a distinct role in signaling and pathogen recognition. Molecular Plant–Microbe Interactions 31: 906–913. 29663867 10.1094/MPMI-12-17-0313-R

[nph17789-bib-0026] Konrad KR , Hedrich R . 2008. The use of voltage‐sensitive dyes to monitor signal‐induced changes in membrane potential‐ABA triggered membrane depolarization in guard cells. The Plant Journal 55: 161–173. 18363788 10.1111/j.1365-313X.2008.03498.x

[nph17789-bib-0027] Kuhlbrandt W . 2004. Biology, structure and mechanism of P‐type ATPases. Nature Reviews. Molecular Cell Biology 5: 282–295. 15071553 10.1038/nrm1354

[nph17789-bib-0028] Kumar S , Stecher G , Tamura K . 2016. Mega7: molecular evolutionary genetics analysis v.7.0 for bigger datasets. Molecular Biology and Evolution 33: 1870–1874. 27004904 10.1093/molbev/msw054 PMC8210823

[nph17789-bib-0029] Kunze G , Zipfel C , Robatzek S , Niehaus K , Boller T , Felix G . 2004. The N terminus of bacterial elongation factor Tu elicits innate immunity in Arabidopsis plants. Plant Cell 16: 3496–3507. 15548740 10.1105/tpc.104.026765 PMC535888

[nph17789-bib-0031] Lee H‐Y , Mang H , Choi E , Seo Y‐E , Kim M‐S , Oh S , Kim S‐B , Choi D . 2021. Genome‐wide functional analysis of hot pepper immune receptors reveals an autonomous NLR clade in seed plants. New Phytologist 229: 532–547. 32810286 10.1111/nph.16878 PMC7756659

[nph17789-bib-0032] Liu J , Elmore JM , Fuglsang AT , Palmgren MG , Staskawicz BJ , Coaker G . 2009. RIN4 functions with plasma membrane H^+^‐ATPases to regulate stomatal apertures during pathogen attack. PLoS Biology 7: e1000139. 19564897 10.1371/journal.pbio.1000139 PMC2694982

[nph17789-bib-0033] Liu Y , Burch‐Smith T , Schiff M , Feng S , Dinesh‐Kumar SP . 2004. Molecular chaperone Hsp90 associates with resistance protein N and its signaling proteins SGT1 and Rar1 to modulate an innate immune response in plants. Journal of Biological Chemistry 279: 2101–2108. 14583611 10.1074/jbc.M310029200

[nph17789-bib-0034] Liu Y , Schiff M , Dinesh‐Kumar SP . 2002. Virus‐induced gene silencing in tomato. The Plant Journal 31: 777–786. 12220268 10.1046/j.1365-313x.2002.01394.x

[nph17789-bib-0035] Maekawa T , Kufer TA , Schulze‐Lefert P . 2011. NLR functions in plant and animal immune systems: so far and yet so close. Nature Immunology 12: 818–826. 10.1038/ni.2083 21852785

[nph17789-bib-0036] Mang H , Feng B , Hu Z , Boisson‐Dernier A , Franck CM , Meng X , Huang Y , Zhou J , Xu G , Wang T *et al*. 2017. Differential regulation of two‐tiered plant immunity and sexual reproduction by ANXUR receptor‐like kinases. Plant Cell 29: 3140–3156. 29150546 10.1105/tpc.17.00464 PMC5757273

[nph17789-bib-0037] Martiniere A , Gibrat R , Sentenac H , Dumont X , Gaillard I , Paris N . 2018. Uncovering pH at both sides of the root plasma membrane interface using noninvasive imaging. Proceedings of the National Academy of Sciences, USA 115: 6488–6493. 10.1073/pnas.1721769115 PMC6016826 29866831

[nph17789-bib-0038] Maudoux O , Batoko H , Oecking C , Gevaert K , Vandekerckhove J , Boutry M , Morsomme P . 2000. A plant plasma membrane H^+^‐ATPase expressed in yeast is activated by phosphorylation at its penultimate residue and binding of 14‐3‐3 regulatory proteins in the absence of fusicoccin. Journal of Biological Chemistry 275: 17762–17770. 10748153 10.1074/jbc.M909690199

[nph17789-bib-0039] Oh S‐K , Kim S‐B , Yeom S‐I , Lee H‐A , Choi D . 2010. Positive‐selection and ligation‐independent cloning vectors for large scale in planta expression for plant functional genomics. Molecules and Cells 30: 557–562. 21340673 10.1007/s10059-010-0156-2

[nph17789-bib-0054] Ortiz D , Dodds PN . 2018. Plant NLR origins traced back to green algae. Trends in Plant Science 23: 651–654. 29887275 10.1016/j.tplants.2018.05.009

[nph17789-bib-0040] Qi Y , Tsuda K , Glazebrook J , Katagiri F . 2011. Physical association of pattern‐triggered immunity (PTI) and effector‐triggered immunity (ETI) immune receptors in Arabidopsis. Molecular Plant Pathology 12: 702–708. 21726371 10.1111/j.1364-3703.2010.00704.x PMC6640369

[nph17789-bib-0041] Saucet SB , Ma Y , Sarris PF , Furzer OJ , Sohn KH , Jones JD . 2015. Two linked pairs of Arabidopsis TNL resistance genes independently confer recognition of bacterial effector AvrRps4. Nature Communications 6: 1–12. 10.1038/ncomms7338 25744164

[nph17789-bib-0042] Schultink A , Qi T , Bally J , Staskawicz B . 2019. Using forward genetics in *Nicotiana benthamiana* to uncover the immune signaling pathway mediating recognition of the *Xanthomonas perforans* effector XopJ4. New Phytologist 221: 1001–1009. 30156705 10.1111/nph.15411

[nph17789-bib-0057] Seo E , Kim S , Yeom SI , Choi D . 2016. Genome‐wide comparative analyses reveal the dynamic evolution of nucleotide‐binding leucine‐rich repeat gene family among Solanaceae plants. Frontiers in Plant Science 7: 1205. 27559340 10.3389/fpls.2016.01205 PMC4978739

[nph17789-bib-0043] Sondergaard TE , Schulz A , Palmgren MG . 2004. Energization of transport processes in plants. Roles of the plasma membrane H^+^‐ATPase. Plant Physiology 136: 2475–2482. 15375204 10.1104/pp.104.048231 PMC523315

[nph17789-bib-0045] Vera‐Estrella R , Barkla BJ , Higgins VJ , Blumwald E . 1994. Plant defense response to fungal pathogens (activation of host‐plasma membrane H^+^‐ATPase by elicitor‐induced enzyme dephosphorylation). Plant Physiology 104: 209–215. 12232073 10.1104/pp.104.1.209 PMC159179

[nph17789-bib-0046] Wan L , Essuman K , Anderson RG , Sasaki Y , Monteiro F , Chung EH , Nishimura EO , DiAntonio A , Milbrandt J , Dangl JL *et al*. 2019. TIR domains of plant immune receptors are NAD(+)‐cleaving enzymes that promote cell death. Science 365: 799–803. 31439793 10.1126/science.aax1771 PMC7045805

[nph17789-bib-0047] Wang J , Hu M , Wang J , Qi J , Han Z , Wang G , Qi Y , Wang HW , Zhou JM , Chai J . 2019. Reconstitution and structure of a plant NLR resistosome conferring immunity. Science 364: eaav5870. 30948527 10.1126/science.aav5870

[nph17789-bib-0048] Win J , Kamoun S , Jones AM . 2011. Purification of effector–target protein complexes via transient expression in *Nicotiana benthamiana* . Methods in Molecular Biology 712: 181–194. 21359809 10.1007/978-1-61737-998-7_15

[nph17789-bib-0049] Xing T , Higgins VJ , Blumwald E . 1996. Regulation of plant defense response to fungal pathogens: two types of protein kinases in the reversible phosphorylation of the host plasma membrane H^+^‐ATPase. Plant Cell 8: 555–564. 12239392 10.1105/tpc.8.3.555 PMC161120

[nph17789-bib-0050] Xing T , Higgins VJ , Blumwald E . 1997. Identification of G proteins mediating fungal elicitor‐induced dephosphorylation of host plasma membrane H^+^‐ATPase. Journal of Experimental Botany 48: 229–237.

[nph17789-bib-0052] Zhou F , Andersen CH , Burhenne K , Fischer PH , Collinge DB , Thordal‐Christensen H . 2000. Proton extrusion is an essential signalling component in the HR of epidermal single cells in the barley–powdery mildew interaction. The Plant Journal 23: 245–254. 10929118 10.1046/j.1365-313x.2000.00777.x

[nph17789-bib-0053] Zuo J , Niu QW , Chua NH . 2000. Technical advance: an estrogen receptor‐based trans activator XVE mediates highly inducible gene expression in transgenic plants. The Plant Journal 24: 265–273. 11069700 10.1046/j.1365-313x.2000.00868.x

