# Supplementary material for: Plasma membrane‐localized plant immune receptor targets H+‐ATPase for membrane depolarization to regulate cell death
Source: New Phytol. 2021 Oct 30;233(2):934–47. doi: 10.1111/nph.17789 (PMC9298278; doi:10.1111/nph.17789)
Supplement: Supplementary file 1 — Fig. S1 Full‐length ANL309 has a capacity to form homodimers. Fig. S2 CCA309 is colocalized with NbPMA3 at the plasma membrane. Fig. S3 CCA309 is associated with NbPMA3, not PM‐localized protein NbPIP2;1. Fig. S4 The silencing efficiency and protein expression in NbPMA3 or NbPMA1/2/3 silencing plants, and expression of NbPMA3 in CCA309 overexpressed plants (Fig. S1). Fig. S5 NbPMA3 is a member of the H+‐ATPase subfamily that plays essential and redundant functions in plant development. Fig. S6 Calibration of the pHluorin response to various external pH buffers. Fig. S7 NbPMA3 compromised the CCA309‐induced apoplastic alkalization. Fig. S8 Nb14‐3‐3 associates with NbPMA3. Fig. S9 The silencing efficiency and protein expression in Nb14‐3‐3 silencing plants. Fig. S10 Full‐length R proteins, CaPvr4 and NbZAR1, associate with NbPMA3. Fig. S11 Proposed molecular mechanism of plasma membrane‐associated CNL‐mediated cell death. Table S1 Primer sequences used in this study. Table S2 List of the CCA309 interactor candidates identified by MS. Please note: Wiley Blackwell are not responsible for the content or functionality of any Supporting Information supplied by the authors. Any queries (other than missing material) should be directed to the New Phytologist Central Office. [file NPH-233-934-s001.pdf]

New Phytologist **Supporting Information**

Article title: **Plasma membrane-localized plant immune receptor targets H^+-ATPase for membrane depolarization to regulate cell death**

Authors: Hye-Young Lee[1*], Ye-Eun Seo[1,2*], Joo Hyun Lee[1], So Eui Lee[1,2], Soohyun Oh[1,2], Jihyun Kim[1,2], Seungmee Jung[1], Haeun Kim[1,2], Hyojeong Park[1,2], Sejun Kim[1,2], Hyunggon Mang[1, *,†], and Doil Choi[1,2†]

Article acceptance date: 22 September 2021

Table. S1 Primer sequences used in this study

Name	Primer (5'-3')	Description
**For cloning**		
CCA309-AF	CGACGACAAGACCCTATGGCTGTGGAAATCA TAACTGC	For overexpression of CC^{A}309 with C-terminal epitope tag
CC^{A}309-AR	GAGGAGAAGAGCCCTAACCGGATAGCAGAA AACAGC	For overexpression of CC^{A}309 with C-terminal epitope tag
NbPIP2;1_AF	CGACGACAAGACCCTATGGCAAAGGATGTG GAAGCCG	For overexpression of NbPIP2;1 with C-terminal epitope tag
NbPIP2;1_AR	GAGGAGAAGAGCCCTGACGTTGGCAGCACTT CTGAATGATC	For overexpression of NbPIP2;1 with C-terminal epitope tag
NbPMA3-AF	CGACGACAAGACCCTATGGGAGAGAAGCCT GAAGTATTG	For overexpression of NbPMA3 with C-terminal epitope tag
NbPMA3-AR	GAGGAGAAGAGCCCTAACGGTGTATGACTGC TGAATTGTC	For overexpression of NbPMA3 with C-terminal epitope tag
NbPMA1-AF	CGACGACAAGACCCTATGGGGGAGGAGAAG CCTG	For overexpression of NbPMA1 with C-terminal epitope tag
NbPMA1-AR	GAGGAGAAGAGCCCTAACAGTGTATGCCTGT TGAATTGTC	For overexpression of NbPMA1 with C-terminal epitope tag
NbPMA4-AF	CGACGACAAGACCCTATGGCAAAAGCTATCA GCCTCG	For overexpression of NbPMA4 with C-terminal epitope tag
NbPMA4-AR	GAGGAGAAGAGCCCTAACTGTATAATGCTGC TGGATCG	For overexpression of NbPMA4 with C-terminal epitope tag
CC^{A}309_attB_F	GGGGACAAGTTTGTACAAAAAAGCAGGCTAT GGCTGTGGAAATCATAACTGCTATT	For cloning into pDONR207
CC^{A}309_attB_R	GGGGACCACTTTGTACAAGAAAGCTGGGTTA ACCGGATAGCAGAAAACAGC	For cloning into pDONR207
NbPMA3-N-AF	CGACGACAAGACCCTATGGGAGAGAAGCCT GAAGTATTG	For overexpression of N-terminal domain of PMA3 with epitope
NbPMA3-N-AR	GAGGAGAAGAGCCCTTTTCAAGAATTTGCTC TCCTTCTTCTCC	For overexpression of N-terminal domain of PMA3 with epitope

NbPMA3-M-AF	CGACGACAAGACCCTATGTCTCATCGTCTTGCTCAACAGGGC	For overexpression of central domain of PMA3 with epitope
NbPMA3-M-AR	GAGGAGAAGAGCCCTCGTGTAGTTTTTCATCCTTTGAAAGATTGC	For overexpression of central domain of PMA3 with epitope
NbPMA3-C-AF	CGACGACAAGACCCTATGAAGTTCTTCATCCGTTATGCTCTCAGTG	For overexpression of C-terminal domain of PMA3 with epitope
NbPMA3-C-AR	GAGGAGAAGAGCCCTAACGGTGTATGACTGCTGAATTGTCTC	For overexpression of C-terminal domain of PMA3 with epitope
CC^{A}309_RI_F	CAGAATTCATGGCTGTGGAAATCATAACTGC	For cloning into pGBKT7 (yeast bait vector)
CCA309_BH_R	CAGGATCCTCAACATATCCCAACAATAGTAATC	For cloning into pGBKT7 (yeast bait vector)
NbPMA3_N_EcoRI_SmaI_F	CAGAATTCCCCGGGGGAGAGAAGCCTGAAGTATTG	For cloning into pGADT7 (yeast prey vector)
NbPMA3_N_SacI_BH_w/stop_R	CAGAGCTCGGATCCTCATTTCAAGAATTTGCTCTCCTTCTTCTCC	For cloning into pGADT7 (yeast prey vector)
NbPMA3_M_EcoRI_SmaI_F	CAGAATTCCCCGGGTCTCATCGTCTTGCTCAACAGGGC	For cloning into pGADT7 (yeast prey vector)
NbPMA3_M_SacI_BH_w/stop_R	CAGAGCTCGGATCCTCACGTGTAGTTTTTCATCCTTTGAAAGATTGC	For cloning into pGADT7 (yeast prey vector)
NbPMA3_C_EcoRI_SmaI_F	CAGAATTCCCCGGGAAGTTCTTCATCCGTTATGCTCTCAGTG	For cloning into pGADT7 (yeast prey vector)
NbPMA3_C_SacI_BH_w/stop_R	CAGAGCTCGGATCCTCAAACGGTGTATGACTGCTGAATTGTCTC	For cloning into pGADT7 (yeast prey vector)
Nb14-3-3_AF	CGACGACAAGACCCTATGGCGGTGGCACCGACG	For overexpression of Nb14-3-3 with C-terminal epitope tag
Nb14-3-3_AR	GAGGAGAAGAGCCCTATTTTTGGCTTCATCAGGTTTGGG	For overexpression of Nb14-3-3 with C-terminal epitope tag
NbPMA3_T955A_AR	GAGGAGAAGAGCCCTTCAAACGGCGTATGACTGCTGAATTGTC	For generation of NbPMA3-T955A mutant
NbPMA3_T955E_AR	GAGGAGAAGAGCCCTTCAAACCTCGTATGACTGCTGAATTGTC	For generation of NbPMA3-T955E mutant
CaPvr4_AF	CGACGACAAGACCCTATGGAAATCGTAACTGCTATTTTGAG	For overexpression of Pvr4 with C-terminal epitope tag
CaPvr4_AR	GAGGAGAAGAGCCCTTCAGATTCGATTCCAATCTTCTCCCTGAG	For overexpression of Pvr4 with C-terminal epitope tag
NbZAR1-AF	CGACGACAAGACCCTATGGTGGATGCGGTGGTCACTG	For overexpression of NbZAR1 with C-terminal epitope tag

NbZAR1-AR	GAGGAGAAGAGCCCTGTTCCTATGTTCTTCC TTCTTCCATAGACC	For overexpression of NbZAR1 with C-terminal epitope tag
CC^{Pvr4}-AF	ATGGAAATCGTAACTGCTATTTTGAGC	For overexpression of CC domain of Pvr4 with C-terminal epitope tag
CC^{Pvr4}-AR	GAGGAGAAGAGCCCTCAAAGCTGTCATGACC TCTTCC	For overexpression of CC domain of Pvr4 with C-terminal epitope tag
CC^{NbZAR1}-AR	GAGGAGAAGAGCCCTATTGTGTGCCTCCATA CTTCCTTCT	For overexpression of CC domain of NbZAR1 with C-terminal epitope tag
CC^{R3a}-AF	CGACGACAAGACCCTATGGAGATTGGCTTAG CAGTTGG	For overexpression of CC domain of R3a with C-terminal epitope tag
CC^{R3a}-AR	GAGGAGAAGAGCCCTAGTCGAACCAAAATG TTCCTTTAAGCC	For overexpression of CC domain of R3a with C-terminal epitope tag
AVR3a_KI-AF	CGACGACAAGACCCTATGGACCAAACCAAG GTCCTGG	For overexpression of Avr3a with C-terminal epitope tag
AVR3a-AR	GAGGAGAAGAGCCCTCATGCATTCCCTATCG ATCTTTATG	For overexpression of Avr3a with C-terminal epitope tag
**For BiFC vector construction**		
YN_F	GCTCTTCTCCTCAGAGAGCAAAAGTTGATTT CTGAGGAGG	For construction of Bi-FC vector
YN_mid R (PstI site removal)	TTGCAGGCCGTAGCCGAAG	For construction of Bi-FC vector
YN_mid Forward (PstI site removal)	TACGGCCTGCAATGCTTCGCCCGCTACCC	For construction of Bi-FC vector
YN_AR	GAGGAGAAGAGCCCTTTAGGCCATGATATAG ACGTTGTGG	For construction of Bi-FC vector
YC_F	GCTCTTCTCCTCATGTACCCATACGATGTTCC AGATTACG	For construction of Bi-FC vector
YC_AR	GAGGAGAAGAGCCCTTTACTTGTACAGCTCG TCCATGCC	For construction of Bi-FC vector
**For qRT-PCR**		
NbPMA1-qRT_F	GTCATTGCTCAACTGGTTGCA	For qRT PCR
NbPMA1-qRT_R	GATGATATCAAGAGGGATATAGAATACTAG	For qRT PCR
NbPMA2-qRT_F	GCCCATGCACAAAGGACT	For qRT PCR
NbPMA2-qRT_R	CAGTGTATGCAGCTCACGTTGC	For qRT PCR

NbPMA3-qRT_F	AGTGATCTGGCTCTACAATATTGTG	For qRT PCR
NbPMA3-qRT_R	CGCTGTTCTTTCCCGAAATCTTTTTTT	For qRT PCR
Nb14-3-3-qRT_F	GCAAAGCTTATCCCATCTGCAGCATC	For qRT PCR
Nb14-3-3-qRT_R	GAGTGTACTCTCAGCAGCCTC	For qRT PCR
**For VIGS**		
TRV-NbPMA3-AF	CGACGACAAGACCCTTCATTACATTGCTTATAATCAACTCCAC	For NbPMA3 silencing construct
TRV-NbPMA3-AR	GAGGAGAAGAGCCCTGATTATGTCCCCAGGCACGAG	For NbPMA3 silencing construct
TRV-NbPMA1/2/3-AF	CGACGACAAGACCCTCCTTTCATGGTGCTTATCATTGCAATTCTTAAT	For NbPMA1/2/3 silencing construct
TRV-NbPMA1/2/3-AR	GAGGAGAAGAGCCCTATTGTAGAGCCAGATCACTCCAGC	For NbPMA1/2/3 silencing construct
TRV-NbPMA1_tR	GCTTCTCCTCCCCCCCAAAAATTTCAAGAATTTGC	For NbPMA1/2/3/4 silencing construct
TRV-NbPMA2_tF	AAATTTTTGGGGTTTATGTGGAATCCTCTCTCATGG	For NbPMA1/2/3/4 silencing construct
TRV-NbPMA2_tR	GGAGCGAGCCGAGCCATG	For NbPMA1/2/3/4 silencing construct
TRV-NbPMA3_tF	TCGGCTCGCTCCGAAAGCCAAGGTCCTTCGAG	For NbPMA1/2/3/4 silencing construct
TRV-NbPMA3_tR	AGACACCATCTCCAGGACC	For NbPMA1/2/3/4 silencing construct
TRV-NbPMA4_tF	GAGATGGTGTCTTCTCTGGCTCAACTTGCAAAC	For NbPMA1/2/3/4 silencing construct
TRV-NbPMA4_AR	GAGGAGAAGAGCCCTGTACATGACTATAATCTCAACGAGC	For NbPMA1/2/3/4 silencing construct
TRV-Nb14-3-3-AF	CGACGACAAGACCCTGAGCAAGCTGAGAGGTACGAAG	For 14-3-3 silencing construct
TRV-Nb14-3-3-AR	GAGGAGAAGAGCCCTGATGGGATAAGCTTTGCATCGAGC	For 14-3-3 silencing construct
**For mutagenesis**		
ANL309_D476V_sense	TTATGTCAAAATGCATGTTGTGGTCCGGGACGTG	
ANL309_D476V_antisense	CACGTCCCGGACCACAACATGCATTTTGACATAA	
NbZAR1_D481V_sense	CACATGCAAAATGCATGTCATGGTTCGTGACTTGG	
NbZAR1_D481V_antisense	CCAAGTCACGAACCATGACATGCATTTTGCATGTG	

Table. S2 List of the CC^{A}309 interactor candidates identified by mass spectrometry

Gene description	Gene ID	# of peptides[a]		Co-IP[b]
		35S:GFP-3xFLAG	35S:CC^{A}309-3xFLAG	
CC^{A}309		0	223	
GFP		1352	0	
ATP synthase epsilon chain	Niben101Scf01399g00012.1	2	30	+
Acetyl-CoA carboxylase	Niben101Scf04077g03006.1	0	26	-
Cell division protein FtsZ homolog 1	Niben101Scf03107g01011.1	0	24	n.t
Chaperonin 60	Niben101Scf02842g00014.1	0	21	-
Glutathione S-transferase	Niben101Scf08250g03016.1	1	21	n.t
EPSIN2	Niben101Scf01066g01009.1	2	18	n.t
Peptidyl-prolyl cis-trans isomerase FKBP16-3	Niben101Scf06423g01016.1	0	18	+
Cinnamoyl-CoA reductase 2-like	Niben101Scf09883g01010.1	0	16	-
Acyl-CoA-binding protein	Niben101Scf02240g02008.1	0	13	n.t
Thioredoxin superfamily protein	Niben101Scf10490g00011.1	0	12	-
Thiamine pyrophosphate enzyme	Niben101Scf02538g06008.1	0	11	-
Coiled coil domain containing protein	Niben101Scf10747g00018.1	0	10	n.t
Chaperonin 60-2	Niben101Scf29076g00011.1	0	10	-
Macrophage migration inhibitory factor	Niben101Scf00302g02011.1	0	10	-
Reactive Intermediate Deaminase A	Niben101Scf04339g07004.1	0	9	+
ATP synthase delta chain	Niben101Scf15372g00003.1	0	8	-
Translation elongation factor EF1B	Niben101Scf03350g00002.1	0	7	-
Trigalactosyldiacylglycerol 2	Niben101Scf07377g00001.1	0	7	+
pre-mRNA-processing factor 39	Niben101Scf11535g01033.1	0	7	n.t
2-methylene-furan-3-one reductase	Niben101Scf01155g00008.1	0	7	-
Plasma membrane ATPase 3	Niben101Scf07395g00031.1	0	6	+
Peroxiredoxin	Niben101Scf03671g02013.1	0	6	-
Superoxide dismutase	Niben101Scf04451g00026.1	0	5	-
Tripeptidyl-peptidase 2 isoform	Niben101Scf00595g08041.1	0	5	n.t
Light-harvesting complex II proteins	Niben101Scf06008g00011.1	0	4	+
Zinc finger A20 and AN1 domain-containing protein	Niben101Scf05348g04001.1	0	3	-

[a]: The number of unique peptides identified is listed.
[b]: Each protein was tested to validate the association with CC^{A}309 by co-immunoprecipitation. This experiment was not repeated (+; association, -; not association, n.t; not tested).

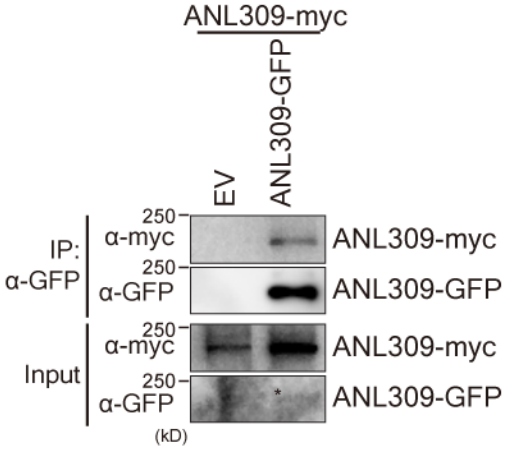

Fig. S1 Full-length ANL309 has a capacity to form homodimer.

ANL309 self-associates *in vivo*. *ANL309-myc* was co-expressed with EV or *ANL309-GFP* in *N. benthamiana*. At 2 days after infiltration, leaves were collected and proteins extracts were immunoprecipitated with α-GFP (IP:α-GFP) and immunoblotted with α-myc or α-GFP (top two panels). Protein inputs are shown with immunoblotting before IP (bottom two panels). Asterisk indicates the expected sizes of proteins.

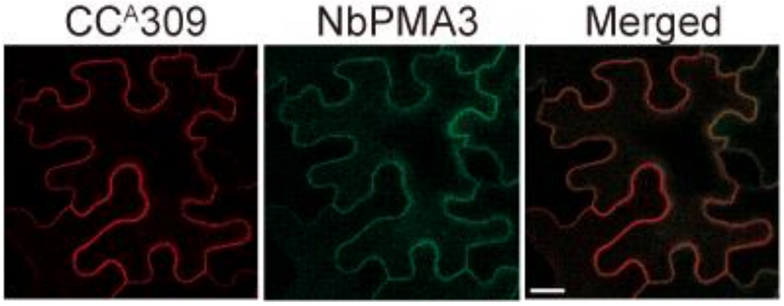

Fig. S2 CC^{A}309 is co-localized with NbPMA3 at plasma membrane.

The subcellular co-localization of the CC^{A}309 and NbPMA3 was determined in *N. benthamiana* epidermal cells. The *Agrobacterium* carrying *CC^{A}309-mstrawberry* or *NbPMA3-eGFP* were co-infiltrated in 4-week-old *N. benthamiana*. At 2 dpi, the fluorescence signals were observed by confocal microscope. The images were obtained by the combination of Z-stack overlays. Bar=20 μm.

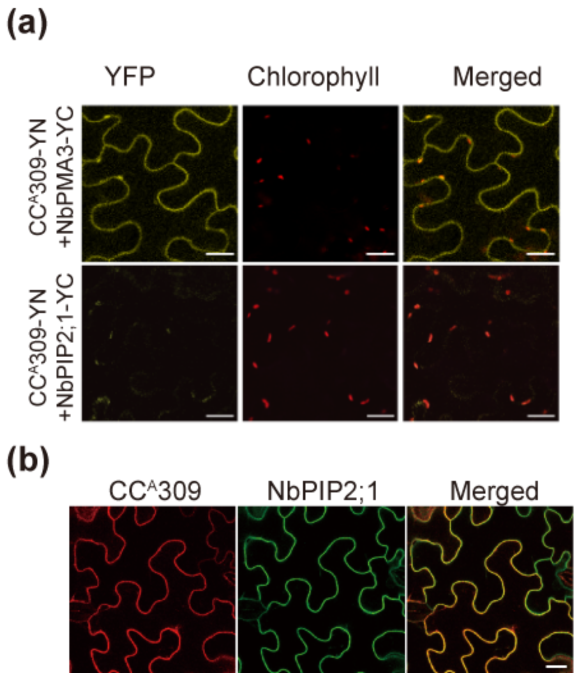

Fig. S3 CC^{A}309 associated with NbPMA3, not PM-localized protein NbPIP2;1.

(a) CC^{A}309 associates with NbPMA3 in BiFC assay, NbPMA3-YC was co-expressed with CC^{A}309-YN or NbPIP2;1-YN. The YFP signals were detected by a confocal microscope after 40 hours post-agro infiltration (hpi). YFP, yellow fluorescent protein signal; Chlorophyll, autofluorescence signal; Merged, YFP merged with Chlorophyll. Scale Bar = 20 μm. NbPIP2;1 localized in PM is used as a negative control.

(b) CC^{A}309 and NbPIP2;1 are co-localized in the plasma membrane. the subcellular co-localization was determined in *N. benthamiana* epidermal cells. CC^{A}309-mstrawberry was co-expressed with NbPIP2;1-eGFP in *N. benthamiana*. The fluorescence signals were observed by confocal microscopy at 2dpi. Bar = 20 μm.

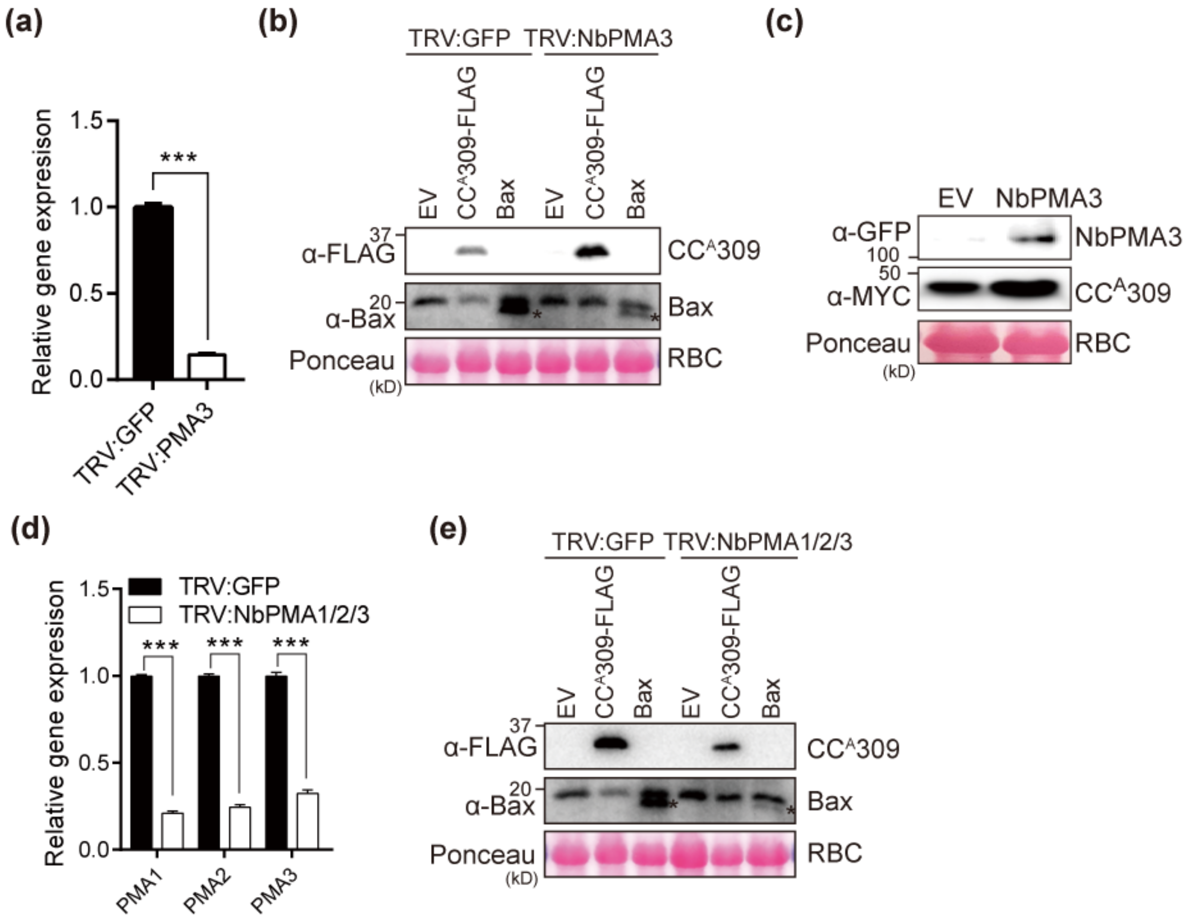

Fig. S4 The silencing efficiency and protein expression in NbPMA3 or NbPMA1/2/3 silencing plants, and the expression of NbPMA3 in CC^{A}309 over-expressed plants (Supports Fig. S1). (**a**) Decreased transcript levels of *NbPMA3* in *NbPMA3*-silenced plant. The transcript levels of *NbPMA3* were measured by quantitative RT-PCR at 2 weeks after VIGS. The mean values (± SD) for transcript levels were normalized to that of *N. benthamiana EF1-α*. Transcript levels of *NbPMA3* in *GFP*-silenced plants were set to 1. Total RNA was extracted from three biological replicate plants. Bar and error bars represent the mean (± SD) of three technical replicates. The mean values for transcript levels were normalized to that of *N. benthamiana EF1-α*. Asterisks denote significant differences at ***P < 0.001 as determined by the t-test. (Supports Fig. 1b)

(**b**) The protein expression of CC^{A}309 and Bax in *NbPMA3*-silenced plants. Silenced plants by *TRV:GFP* or *TRV:NbPMA3* were agro-infiltrated with EV, *CC^{A}309-FLAG*, or *Bax* at two weeks after silencing. Protein accumulation in *GFP*- or *PMA3*-silenced plants was confirmed by immunoblot with α-FLAG or –BAX antibody at 3 dpi (top two panels). Equal protein loading was confirmed by Ponceau S staining of membrane (bottom panel). Asterisks indicate the expected protein bands. (Supports Fig. 1b)

(**c**) The protein expression of NbPMA3 in CC^{A}309 expressed plants. *CC^{A}309-MYC* was co-expressed *NbPMA3-GFP* or EV in *N. benthamiana*. The proteins were detected by

immunoblotting with α-GFP or α-MYC at 3dpi. Ponceau S staining of RuBisCO (RBC) is loading control (left bottom). (Supports Fig.1c).

(**d**) The knock-down transcripts levels of *NbPMA1*, *NbPMA2*, and *NbPMA3* in *PMA1/2/3*-silenced plants. The transcript levels were measured by qRT-PCR. Total RNA was extracted from three biological replicate plants. Bar and error bars represent the mean (± SD) of three technical replicates. The mean values for transcript levels were normalized to that of *N. benthamiana EF1-α*. Asterisks denote significant differences at ***P < 0.001 as determined by the t-test. (Supports Fig. 1e)

(**e**) The protein expression of CC^{A}309 and Bax in NbPMA1/2/3-silenced plants. Silenced plants by *TRV:GFP* or *TRV:NbPMA1/2/3* were agro-infiltrated with EV, *CC*^{A}*309-FLAG*, or *Bax* at two weeks after silencing. Protein accumulation in *GFP*- or *PMA3*-silenced plants was confirmed by immunoblot with α-FLAG or α-BAX antibody at 3 dpi (top two panels). Equal protein loading was confirmed by Ponceau S staining of membrane (bottom panel). Asterisks indicate the expected protein bands. (Supports Fig. 1e)

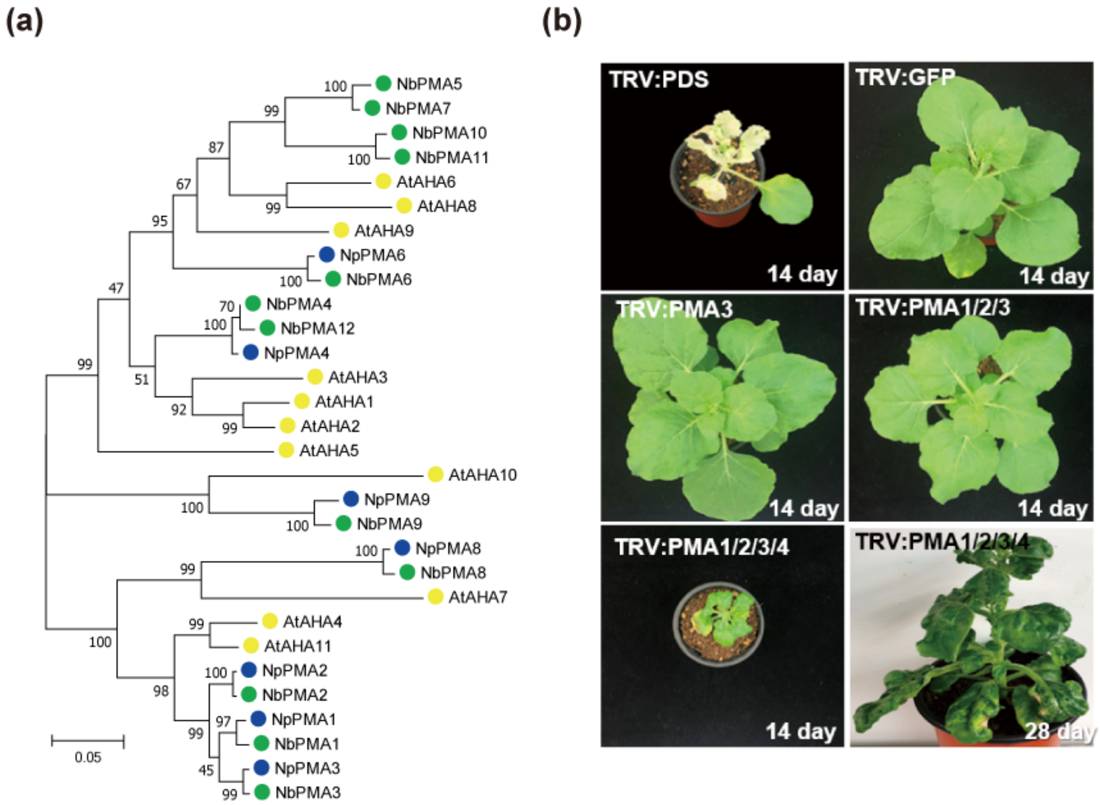

Fig. S5 NbPMA3 is a member of the H^{+}-ATPases subfamily that plays essential and redundant functions for plant development.

(**a**) Phylogenetic analysis of *PMAs* in *Arabidopsis*, *N. benthamiana* and *N. plumbaginifolia*. Amino acid sequences of 11 *PMAs* in *Arabidopsis thaliana*, 12 *PMAs* in *N. benthamiana*, and 9 *PMAs N. plumbaginifolia* were aligned for the construction of the phylogenetic tree. Yellow; *Arabidopsis* PMAs; blue, *N. plumbaginifolia*; green, *N. benthamiana*. Phylogenetic analysis was conducted by a maximum likelihood method with 500 replications of bootsrapping in MEGA7.

(**b**) The phenotypes of *NbPMA3*-, *NbPMA1/2/3*- or *NbPMA1/2/3/4*-silenced plants. Abnormal growth phenotypes were observed in *PMA1/2/3/4*-silenced plant. The photobleached leaves of *phytoene desaturase* (*PDS*)-silenced plant represents the effective gene silencing. The *GFP*-silenced plant was used as a control for VIGS. Pictures were taken at 14 or 28 days after VIGS.

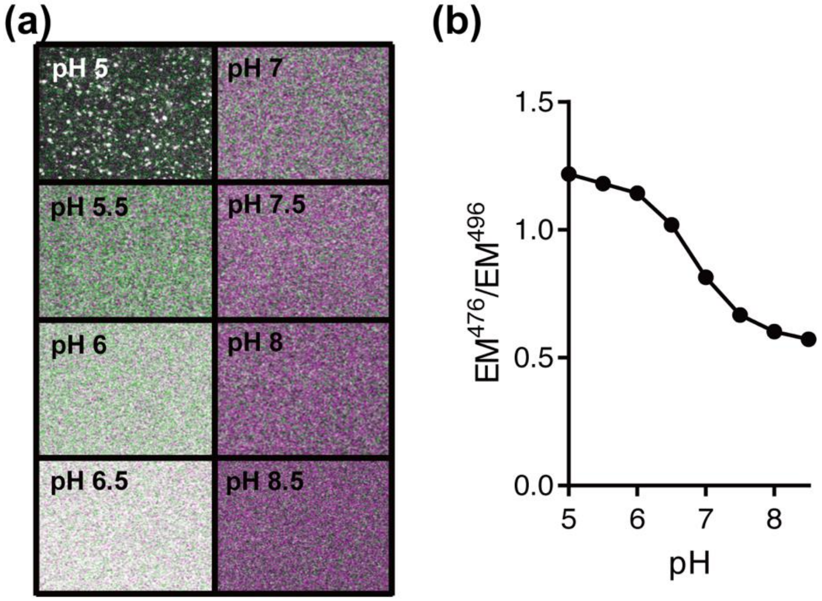

Fig. S6 Calibration of the pHluorin response to various external pH buffers.

(a) pHluorin response to external pH and Calibration curve. Pseudocolored fluorescence images of pHluorin in buffers with different pH values. The purified pHluorin proteins from *E. coli* liquid culture were diluted in 50 mM MES-KOH buffers at pH5, 5.5, 6, 6.5 or in 50 mM HEPES-KOH at pH 7, 7.5, 8, 8.5. The buffers containing the pHluorin dropped onto slide glass and illuminated at 476 nm and 496 nm using a confocal microscope.

(**b)** Calibration curve was obtained from (a) to define absolute pH values. pH was calculated by fitting experimental points with a sigmoid function.

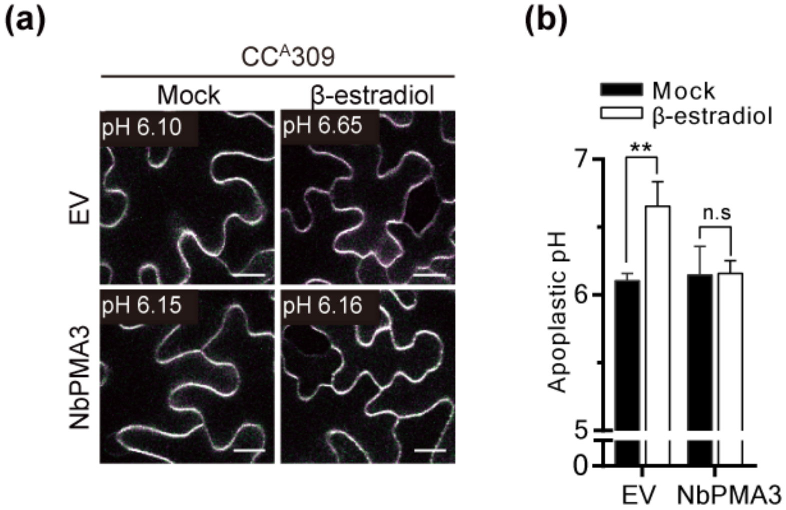

Fig. S7 NbPMA3 compromised the CC^A309-induced apoplastic alkalization.

PM-APO was co-expressed with EV or *NbPMA3-FLAG*. The confocal images (a) and the pH values (b) were taken 4 hr after β-estradiol treatment. The data are shown as mean ± SD (N = 7) by triplicated independent experiments. Asterisk denotes a significant difference (t-test, $^{**}P < 0.01$). Bar=15 μm.

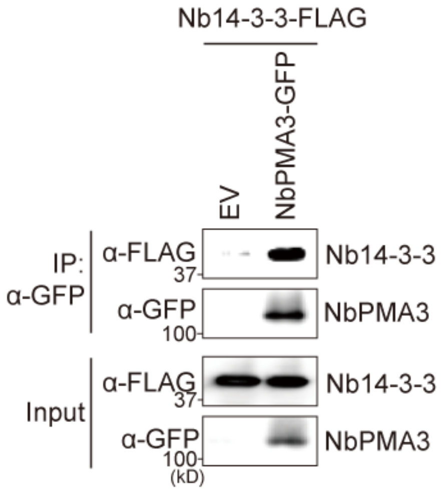

Fig. S8 Nb14-3-3 associates with NbPMA3.

Nb14-3-3 associates with NbPMA3. *14-3-3-FLAG* was co-expressed with EV and *NbPMA3-GFP*. Proteins extracts were immunoprecipitated with α-GFP (IP:α-GFP) and immunoblotted with α-FLAG or α-GFP (top two panel). Protein inputs are shown with immunoblotting before IP (bottom two panels).

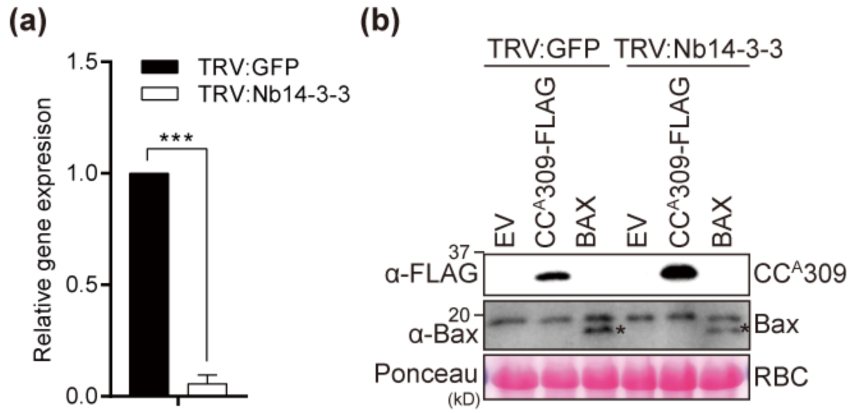

Fig. S9 The silencing efficiency and protein expression in Nb14-3-3 silencing plants.
(a) Decreased transcript levels of *Nb14-3-3* in *Nb14-3-3*-silenced plant. The transcript levels of Nb*14-3-3* were determined by qRT-PCR at 2 weeks after VIGS. Total RNA was extracted from three biological replicate plants. Bar and error bars represent the mean (± SD) of three technical replicates. The mean values for transcript levels were normalized to that of *N. benthamiana EF1-α*. Transcript levels Nb*14-3-3* in *GFP*-silenced plants were set to 1. Asterisks denote significant differences at ***$P < 0.001$ as determined by the t-test.
(b) Protein accumulation of CC^{A}309 and Bax in *GFP*- or *14-3-3*-silenced plants. The expression levels of *CC^{A}309* and *Nb14-3-3* were confirmed by western blot with α-FLAG or α-Bax antibody (top two panels) at 3 dpi. Equal protein loading was confirmed by Ponceau S staining of membrane (bottom panel). Asterisks indicate the expected protein bands.

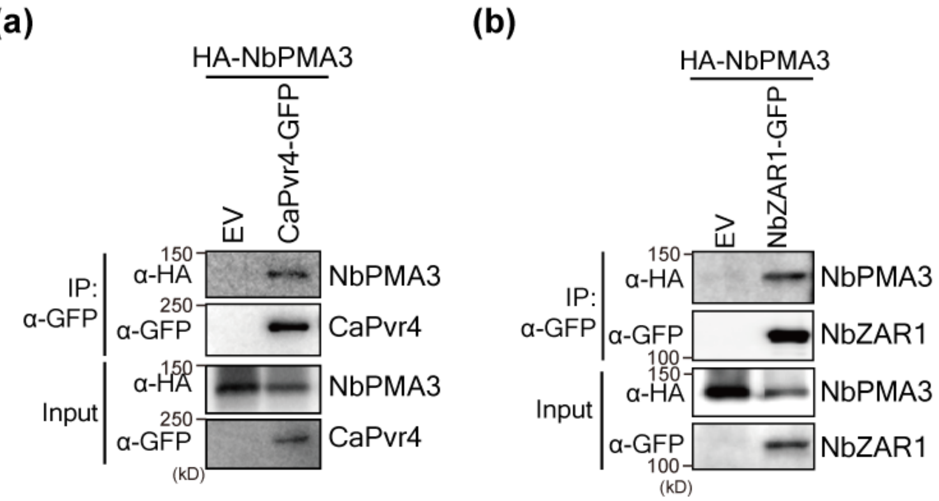

Fig. S10 Full-length R proteins, CaPvr4 and NbZAR1 associate with NbPMA3.

(a) NbPMA3 interacts with CaPvr4. *HA-PMA3* was co-expressed with EV or *CaPvr4-GFP* in *N. benthamiana*. Proteins extracts were immunoprecipitated with α-GFP (IP:α-GFP) and immunoblotted with α-HA or α-GFP (top two panels). Protein inputs are shown with immunoblotting before IP (bottom two panels).

(b) NbPMA3 interacts with NbZAR1. *HA-PMA3* was co-expressed with EV or *CaNbZAR1-GFP* in *N. benthamiana*. Proteins extracts were immunoprecipitated with α-GFP (IP:α-GFP) and immunoblotted with α-HA or α-GFP (top two panels). Protein inputs are shown with immunoblotting before IP (bottom two panels).

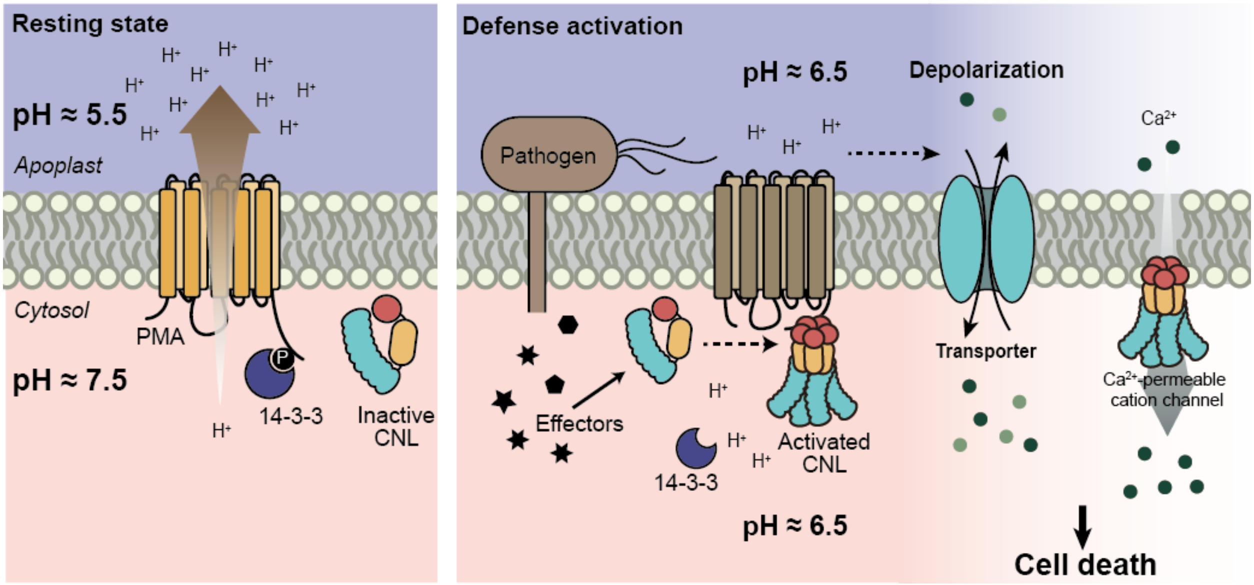

Fig. S11 Proposed molecular mechanism of plasma membrane (PM)-associated CNL-mediated cell death.

The resting state of polarized plasma membrane. The phosphorylation of the penultimate residue of PMA recruits the 14-3-3, resulting in the activation of PMA. Eventually, the proton gradient is formed across the plasma membrane to keep proton homeostasis in the cells (left panel). The perception of effectors derived from various pathogens triggers activation of PM-associated CNLs leading to the association with inactive PMA at their cytosolic central and C-terminal regions. This results in inhibiting phosphorylation of PMA and keep from inactive PMA to active PMA. It disturbs the proton gradient across the PM, leading to PM depolarization. The change of PM potential likely facilitates a series of PM-integrated defense responses such as Ca^{2+} influx. Recently, *Arabidopsis* ZAR1 and helper NLRs, NRG1 and ADR1, have been reported to oligomerize themselves. The multimeric complex functions as a calcium channel to regulate cytosoplasmic [Ca^{2+}], leading to cell death. The cooperation between the change of PM potential and Ca^{2+} influx by NLR resistosome remains to be elucidated. Solid arrow indicates established interactions; dashed arrows represent reactions we proposed in this study.
